# A Location‐Scale Joint Model for Studying the Link Between the Time‐Dependent Subject‐Specific Variability of Blood Pressure and Competing Events

**DOI:** 10.1002/sim.70244

**Published:** 2025-09-05

**Authors:** Léonie Courcoul, Christophe Tzourio, Mark Woodward, Antoine Barbieri, Hélène Jacqmin‐Gadda

**Affiliations:** ^1^ Univ. Bordeaux, INSERM, Bordeaux Population Health, U1219 France; ^2^ The George Institute for Global Health Imperial College London UK; ^3^ The George Institute for Global Health University of New South Wales Sydney Australia

**Keywords:** blood pressure, cardio and cerebrovascular diseases, competing events, heterogeneous variance, joint model, location‐scale model

## Abstract

Given the high incidence of cardio and cerebrovascular diseases (CVD), and their association with morbidity and mortality, their prevention is a major public health issue. A high level of blood pressure is a well‐known risk factor for these events, and an increasing number of studies suggest that blood pressure variability may also be an independent risk factor. However, these studies suffer from significant methodological weaknesses. In this work, we propose a new location‐scale joint model for the repeated measures of a marker and competing events. This joint model combines a mixed model including a subject‐specific and time‐dependent residual variance modeled through random effects, and cause‐specific proportional intensity models for the competing events. The risk of events may depend simultaneously on the current value of the variance, as well as, the current value and the current slope of the marker trajectory. The model is estimated by maximizing the likelihood function using the Marquardt–Levenberg algorithm. The estimation procedure is implemented in an R‐package and is validated through a simulation study. This model is applied to study the association between blood pressure variability and the risk of CVD and death from other causes. Using data from a large clinical trial on the secondary prevention of stroke, we find that the current individual variability of blood pressure is associated with the risk of CVD and death. Moreover, the comparison with a model without heterogeneous variance shows the importance of taking into account this variability in the goodness‐of‐fit and for dynamic predictions.

## 1 Introduction

Cardiovascular diseases, such as ischaemic heart disease, and cerebrovascular events are two leading causes of death. Moreover, these diseases often lead to acquired physical disability or to dementia. Medical care and disability management following this type of disease generate significant societal, human, and financial distress [1]. Given the frequency of cardio and cerebrovascular diseases (CVD) and their dramatic consequences at the individual and societal level, the identification of modifiable risk factors is essential to implement prevention programs. Hypertension (high values of blood pressure) is a well‐known risk factor for these diseases. More recently, some studies have suggested that the visit‐to‐visit variability of blood pressure could be associated with an increased risk of stroke and cardiovascular events independently of the level of blood pressure [2, 3, 4]. These studies have used the individual empirical standard deviation, or some other measure of variation (e.g., the coefficient of variation) or extreme value (e.g., the maximum), of blood pressure as an explanatory variable in a Cox model for the event risk. However, they were exposed to methodological issues. A first strategy consists of calculating the empirical standard deviation of blood pressure on all available measurements [5]. This strategy induces conditioning on the future, likely leading to bias because measurements after the current time (and sometimes after the event time) are used to predict the event at the current time [6, 7]. A second strategy consists of computing the standard deviation of blood pressure on the measurements collected over an initial period of the study, keeping in the sample only the individuals who did not have the event before the end of this period in order to predict the risk beyond this period [7]. This could induce selection bias and certainly creates loss of power. To avoid these issues, the standard deviation of blood pressure can be considered as a time‐dependent variable and calculated using only measurements before the event. Nevertheless, this approach neglects the measurement error of the standard deviation, which is a serious issue when the number of measurements differs between individuals, and requires imputation of the standard deviation at all event times. These limitations may introduce bias [8].

Recently, De Courson et al. [7] compared these different approaches and obtained contradictory results depending on the estimator used for the variability. Furthermore, they pointed out that blood pressure and its standard deviation are endogenous variables for which the Cox model is not suitable [9]. Thus, they also considered a joint model approach combining a mixed model to fit individual trajectories of standard deviation of blood pressure measurements and a proportional hazard model to assess the impact of the current variability on the event risk. In the joint model, the current value of standard deviation of blood pressure is included as a time‐dependent explanatory variable in the time‐to‐event model. This allows the evaluation of the impact of the longitudinal data on the event risk without bias, contrary to the two stage estimation [10, 11, 12]. However, the interpretation of the association between the event risk and the variability computed as the standard deviation of measurements observed since the beginning is difficult. Indeed, this global variability encompass the individual time trend of the blood pressure while the clinical question is the following: is irregularity among blood pressure measures a risk factor independently of current values and possibly time‐trend of blood pressure?

Location‐scale mixed models have been introduced to investigate the heterogeneity of intra‐subject variability for longitudinal data by introducing random effects in the residual variance modeling [13]. For studying the association between the variability of a biomarker and a clinical event, Gao et al. [14] and Barrett et al. [15] have proposed a joint model combining a mixed model including a subject‐specific random effect for the residual variance and a proportional hazard model for the event risk. However, the considered dependence structure is quite restrictive since, in their models, the event risk depends only on the random effects and not on time‐dependent characteristics of the marker trajectory, such as the current value or the current slope. In addition, none of them consider time‐dependent subject‐specific variability of the maker and they do not handle competing events. However, it is essential to account for competing death from other causes because mortality and cardiovascular risk increase with age and may be both associated with blood pressure.

The objective of our work was, therefore, to propose a new location‐scale joint model accounting for both time‐dependent individual variability of a marker and competing events. To do this, we extended the model proposed by Gao et al. [14] and Barrett et al. [15] to include a time‐dependent variability, competing events, a more flexible dependence structure between the event and the marker trajectory, and more flexible baseline risk functions. In contrast to the previous works we propose a frequentist estimation approach which is implemented in the R‐package LSJM.

This paper is organized as follows. Section 2 describes the model and the estimation procedure using a robust algorithm for maximizing the likelihood. Section 3 presents a simulation study to assess the estimation procedure performance. In Section 4, the model is applied to the data from the Perindopril Protection Against Stroke Study (PROGRESS) clinical trial, a blood‐pressure lowering trial for the secondary prevention of stroke [16]. Finally, Section 5 concludes this work with some elements of discussion.

## 2 Method

Let us consider a sample of N individuals. For each individual i∈{1,…,N}, we consider the $n_{i}$‐vector of repeated measures $Y_{i}={\left(Y_{i1},\ldots ,Y_{in_{i}}\right)}^{⊤}$ with $Y_{ij}$ the value of the longitudinal outcome of individual i at time $t_{ij}\;(j=1,\ldots ,n_{i})$. Assuming K competing events, we denote $T_{i}=\min(C_{i},T_{ik}^{*},k=1,\ldots ,K)$ the observed time with $T_{ik}^{*}$ the real time for the event k(k=1,…,K) and $C_{i}$ the censoring time for the ith individual. We then denote $\delta_{i}\in \{0,1,\ldots ,K\}$ the individual event indicator such as $\delta_{i}=k$ if the competing event k∈{1,…,K} occurs and $\delta_{i}=0$ otherwise.

### 2.1 Joint Model With Time‐Dependent Individual Variability

We propose joint modeling for a longitudinal outcome and competing events using a shared random‐effect approach. Joint models allow simultaneous analysis of longitudinal data and clinical events. They combine a mixed model for repeated measures of exposure and a time‐to‐event models. Functions of the random effects from the mixed model are included as explanatory variables in the time‐to‐event models to account for the association between the two outcomes. The longitudinal submodel is defined by a linear mixed‐effect model with heterogeneous variance:

(1) $$\left\{\begin{array}{l} Y_{ij}=Y_{i}(t_{ij})={\tilde{y}}_{i}(t_{ij})+\epsilon_{ij}=X_{i}{\left(t_{ij}\right)}^{⊤}\beta +Z_{i}{\left(t_{ij}\right)}^{⊤}b_{i}+\epsilon_{i}(t_{ij}),\\ \epsilon_{ij}(t_{ij})∼𝒩(0,\sigma_{i}^{2}(t_{ij}))\;\text{with}\;\log(\sigma_{i}(t_{ij}))=O_{i}{\left(t_{ij}\right)}^{⊤}\mu +M_{i}{\left(t_{ij}\right)}^{⊤}\tau_{i} \end{array}\right.$$

${\tilde{y}}_{i}(t)$ is the value at time t of a continuous time process that represents the true underlying value of the variable Y at any time (without measurement error); $X_{i}(t_{ij})$ and $O_{i}(t_{ij})$ are two vectors of explanatory variables for subject i at visit j, possibly including fixed variables, functions of time, interactions between fixed variables and functions of time and time‐dependent variables assumed to be known at any time; $Z_{i}(t_{ij})$ and $M_{i}(t_{ij})$ are, respectively sub‐vectors of $X_{i}(t_{ij})$ and $O_{i}(t_{ij})$ including a column of 1 and functions of time; β and μ are vectors of fixed effects while $b_{i}$ and $\tau_{i}$ are the vectors of subject‐specific random effects such as

$$\left(\begin{array}{c} b_{i}\\ \tau_{i} \end{array}\right)∼𝒩\left(\left(\begin{array}{c} 0\\ 0 \end{array}\right),\left(\begin{array}{cc} \sum_{b} & \sum_{\tau b}\\ \sum_{\tau b}^{⊤} & \sum_{\tau } \end{array}\right)\right)$$

The risk function for the event k∈{1,…,K} is defined by

(2) $$\lambda_{ik}(t)=\lambda_{0k}(t)\exp\left(W_{ik}^{⊤}\gamma_{k}+\alpha_{1k}{\tilde{y}}_{i}(t)+\alpha_{2k}{\tilde{y}}_{i}^{\prime }(t)+\alpha_{\sigma k}\sigma_{i}(t)\right)$$

with $\lambda_{0k}(t)$ the baseline risk function, $W_{ik}$ a vector of baseline covariates associated with the regression coefficient $\gamma_{k}$. The hazard rates at time t depend on characteristics of the marker trajectory at time t: the underlying value of the marker at the current time ${\tilde{y}}_{i}(t)$, the slope of the marker trajectory at the current time ${\tilde{y}}_{i}^{\prime }(t)=d{\tilde{y}}_{i}(t)/dt$ and/or the individual error variance of the marker at the current time $\sigma_{i}(t)$. These associations are, respectively, measured by parameters $\alpha_{1k}$, $\alpha_{2k}$ and $\alpha_{\sigma k}$.This markovian assumption could be relaxed by introducing a time lag. Different parametric forms for the baseline risk function can be considered, such as exponential, Weibull, or, for more flexibility, a B‐splines base with Q knots defined by:

$$\lambda_{0k}(t)=\exp\left(\overset{Q+4}{\underset{q=1}{\sum }}\eta_{qk}B_{q}(t,\nu_{k})\right)$$

where $B_{q}(t,\nu_{k})$ is the qth basis function of B‐splines with the knot vector $\nu_{k}$ and $\eta_{qk}$ is the associated parameter to be estimated.

### 2.2 Estimation Procedure

Let θ be the set of parameters to be estimated including parameters of the Cholesky decomposition of the covariance matrix of the random effects, β, μ, $\alpha^{⊤}=(\alpha_{11},\alpha_{21},\alpha_{\sigma 1},\ldots ,\alpha_{1K},\alpha_{2K},\alpha_{\sigma K})$, $\gamma^{⊤}=(\gamma_{1}^{⊤},\ldots ,\gamma_{K}^{⊤})$ and the parameters of the K baseline risk functions. Considering the frequentist approach, the parameters are estimated by maximizing the likelihood function. Assuming $T_{i}$ independent of $C_{i}$ given $W_{i}$ and marker trajectory characteristics, the contribution of individual i to the marginal likelihood is defined by:

$$\begin{array}{ll} {ℒ}_{i}(\theta ;Y_{i},T_{i},\delta_{i}) & =\int p(Y_{i},T_{i},\delta_{i}|b_{i},\tau_{i};\theta )f(b_{i},\tau_{i};\theta )db_{i}d\tau_{i}\\ & =\int f(Y_{i}|b_{i},\tau_{i};\theta )\exp\left(-\overset{K}{\underset{k=1}{\sum }}\Lambda_{ik}(T_{i}|b_{i},\tau_{i};\theta )\right)\\ & \;\overset{K}{\underset{k=1}{\prod }}\lambda_{ik}{\left(T_{i}|b_{i},\tau_{i};\theta \right)}^{1_{\delta_{i}=k}}f(b_{i},\tau_{i};\theta )db_{i}d\tau_{i} \end{array}$$

with $f(b_{i},\tau_{i};\theta )$ a multivariate Gaussian density and $f(Y_{i}|b_{i},\tau_{i};\theta )=\prod_{j=1}^{n_{i}}f(Y_{ij}|b_{i},\tau_{i};\theta )$ where $f(Y_{ij}|b_{i},\tau_{i};\theta )$ is a univariate Gaussian density; $\Lambda_{ik}(T_{i}|b_{i},\tau_{i};\theta )$ is the cumulative risk function for event k (k∈{1,…,K}) given by:

(3) $$\Lambda_{ik}(t|b_{i},\tau_{i};\theta )=\int_{0}^{t}\lambda_{ik}(u|b_{i},\tau_{i};\theta )du$$

In cohort studies, data are frequently left‐truncated. Left‐truncation arises as soon as the time scale is not the time since inclusion and the subjects are enrolled only if they are free of the event at inclusion [17]. This is the case in most studies where the time scale is age. To deal with left truncation (also called delayed entry), the individual contribution to the likelihood must be divided by the probability to be free of any event at entry time $T_{0i}$:

$${ℒ}_{i}^{DE}(\theta ;Y_{i},T_{i},\delta_{i})=\frac{{ℒ}_{i}(\theta ;Y_{i},T_{i},\delta_{i})}{\int \exp(-\sum_{k=1}^{K}\Lambda_{ik}(T_{0i}|b_{i},\tau_{i};\theta ))f(b_{i},\tau_{i};\theta )db_{i}d\tau_{i}}$$

Because the integral on the random effects does not have an analytical solution, the integral is computed by a Quasi Monte Carlo (QMC) approximation [18], using deterministic quasi‐random sequences. The approximation of the integral is defined by

$${ℒ}_{i}(\theta ;Y_{i},T_{i},\delta_{i})≃\frac{1}{S}\overset{S}{\underset{s=1}{\sum }}p(Y_{i},T_{i},\delta_{i}|b_{i}^{s},\tau_{i}^{s};\theta )$$

where $\left(b_{i}^{1},\ldots ,b_{i}^{S}\right)$ and $\left(\tau_{i}^{1},\ldots ,\tau_{i}^{S}\right)$ are draws of a S‐sample in the sobol sequel for the distribution $f(b_{i},\tau_{i};\theta )$. Moreover, to approximate the cumulative risk function given in equation (3), we use the Gauss–Kronrod quadrature approximation with 15 points [19].

Parameter estimation is obtained by maximizing the log‐likelihood function $\ell (\theta ;Y_{i},T_{i},\delta_{i})=\log\left(\prod_{i=1}^{N}{ℒ}_{i}(\theta ;Y_{i},T_{i},\delta_{i})\right)$. The maximization is performed using the marqLevAlg R‐package based on the Marquardt–Levenberg algorithm [20]. The latter is a robust variant of the Newton–Raphson algorithm [21, 22], which iteratively updates the parameters θ to be estimated until convergence with the following formula at iteration l+1:

$$\theta^{\left(l+1\right)}=\theta^{\left(l\right)}-\psi_{l}\tilde{H}{\left(\theta^{\left(l\right)}\right)}^{-1}\nabla (\ell (\theta^{\left(l\right)}))$$

where $\theta^{\left(l\right)}$ is the set of parameters at iteration l, $\nabla \left(\ell \left(\theta^{\left(l\right)}\right)\right)$ the gradient of the log‐likelihood at iteration l and $\tilde{H}\left(\theta^{\left(l\right)}\right)$ the inflated Hessian matrix where the diagonal terms of the Hessian matrix $H\left(\theta^{\left(l\right)}\right)$ are replaced by

$$\tilde{H}{\left(\theta^{\left(l\right)}\right)}_{ii}=H{\left(\theta^{\left(l\right)}\right)}_{ii}+\phi_{l}\left[\left(1-\rho_{l}\right)\left|H{\left(\theta^{\left(l\right)}\right)}_{ii}\right|+\rho_{l}\mathrm{tr}\left(\theta^{\left(l\right)}\right)\right]$$

The scalars $\psi_{l}$, $\phi_{l}$, and $\rho_{l}$ are internally determined at each iteration l to ensure that $\tilde{H}\left(\theta^{\left(l\right)}\right)$ be definite‐positive, $\tilde{H}\left(\theta^{\left(l\right)}\right)$ approaches $H\left(\theta^{\left(l\right)}\right)$ when θ approaches $\hat{\theta }$ and insure improvement of the likelihood at each iteration. Stringent convergence criteria are used, relying on parameter and function stability, and the relative distance to the maximum computed from the first and second derivatives of the log‐likelihood which must not exceed a threshold $\varepsilon_{d}:\frac{\nabla \left(\ell \left(\theta^{\left(l\right)}\right)\right){\left(H\left(\theta^{\left(l\right)}\right)\right)}^{-1}\nabla \left(\ell \left(\theta^{\left(l\right)}\right)\right)}{m}<\varepsilon_{d}$, with m the number of parameters. This algorithm was previously compared to other algorithms (EM, BFGS and L‐BFGS‐B) and the results showed that this algorithm was the most reliable [20]. The variances of the estimates are estimated by the inverse of the Hessian matrix computed by finite differences. The variances of the estimated parameters from the covariance matrix of the random effects are computed using the Delta‐Method [23].

In the estimation procedure, the choice of the number of QMC draws is an important factor in practice. Indeed, increasing the number of QMC draws ensures good precision of parameter variances for statistical inference but at the cost of a considerable increase in computation time. To limit computation time and ensure precise estimates of parameters and their standard error, we propose a two‐step strategy. We first applied the Marquardt algorithm with a small number S1 of QMC draws (e.g., S1=500) until convergence is achieved. The first step provides a good parameter estimation in a reasonable time, but does not guarantee the accuracy of their estimated standard errors. In the second step, to improve the accuracy of the computation of the standard error of the estimates, a few additional iterations are performed with a higher number S2>S1 of QMC draws (e.g., S2=5000) until the Hessian matrix is invertible. Note that for model selection based on likelihood or information criteria, using the results obtained in the first step is sufficient, given the good estimation of model parameters. However, for statistical inference on the final model, we recommend increasing the number of QMCs (step 2).

### 2.3 Individual Predictions

We implemented the computation of individual probability of having event k between time s and s+t given that the subject i has not experienced any event before time s, all his/her marker measures collected until time $s\;({𝒴}_{i}(s)=\{Y_{i}(t_{ij})|t_{ij}<s\})$, and the set of estimated parameters. The prediction is defined for subject i by

(4) $$\begin{array}{ll} \pi_{i}(s,t;\hat{\theta }) & =P(s<T_{i}<s+t,\delta_{i}=k|T_{i}>s,Y_{i}(s),\hat{\theta })\\ & =\frac{\int \left[\int_{s}^{s+t}g(u)\lambda_{ik}(u|b_{i},\tau_{i},\hat{\theta })du\right]f(Y_{i}(s)|b_{i},\tau_{i},\hat{\theta })f(b_{i},\tau_{i}|\hat{\theta })db_{i}d\tau_{i}}{\int g(s)(s|b_{i},\tau_{i},\hat{\theta }))f(Y_{i}(s)|b_{i},\tau_{i},\hat{\theta })f(b_{i},\tau_{i}|\hat{\theta })db_{i}d\tau_{i}}\\ \text{with}\; & g(u)=\exp(-\sum_{c=1}^{K}\Lambda_{ic}(u|b_{i},\tau_{i},\hat{\theta })) \end{array}$$

As previously, the integral over the random effect is computed by QMC approximation and the integral over time with the Gauss–Kronrod quadrature.

The corresponding 95% confidence interval of predictions is obtained by the following Monte Carlo algorithm.

For L large enough and l=1,…,L(L=1000for instance):

- Generate ${\tilde{\theta }}^{\left(l\right)}∼𝒩(\hat{\theta },V(\hat{\theta }))$ where $V(\hat{\theta })$ is given by the inverse of the Hessian matrix at $\hat{\theta }$
- Compute ${\tilde{\pi }}_{i}^{\left(l\right)}(s,t;{\tilde{\theta }}^{\left(l\right)})$ from equation (4)
- Compute the 95% confidence interval from the 2.5th and 97.5th percentiles of the L‐sample of ${\tilde{\pi }}_{i}^{\left(l\right)}(s,t;{\tilde{\theta }}^{\left(l\right)})$

### 2.4 Software

The R‐package LSJM has been developed for the estimation of the model, the prediction of the subject‐specific random effects, and the computation of the individual predicted probabilities of events. The package allows estimation of a model with an unconstrained time trend for the marker trajectory, constant or time‐dependent subject‐specific residual variance, one or two events with exponential, Weibull or B‐splines baseline risk functions, and a flexible dependence structure between the events and the marker (possibly including the current value, the current slope and the current subject‐specific residual variance). The development version of LSJM is available on Github at the following link: https://github.com/LeonieCourcoul/LSJM. The package also allows the estimation of the linear mixed effect model with heterogeneous variance (or location‐scale mixed model) without jointly modeling the time‐to‐events. This preliminary analysis is required to obtain initial values for the joint model and check that some random‐effect variances are not null. Indeed a joint model assuming, for instance, a dependence on the random slope or on the random variance would be unidentifiable if the variances of these random effects were null.

## 3 Simulations

In order to evaluate the performance of the estimation procedure, we performed a simulation study using a design similar to the application data. In the next sections, we describe the simulation design according to ADEMP framework [24].

### 3.1 Aim

The aim of our simulation was to assess the good convergence properties of the proposed estimation procedure for the location‐scale joint model. By denoting θ, the whole set of parameters of the location‐scale joint model, we wanted to obtain: (1) consistent and unbiaised estimated parameters $\hat{\theta }$, (2) a consistent estimate of the empirical standard error of $\hat{\theta }$, $\hat{SE}(\hat{\theta })$, and (3) a nominal coverage rate for the 95% confidence interval of $\hat{\theta }$.

### 3.2 Data‐Generating Model

The design of our simulation was based on the application on PROGRESS data, using the following model to generate the data:

(5) $$\left\{\begin{array}{ll} Y(t_{ij}) & =142+b_{0i}+(3+b_{1i})\times t_{ij}+\epsilon_{i}(t_{ij})\\ \epsilon_{i}(t_{ij}) & ∼N(0,\sigma_{i}^{2}(t_{ij}))\;\text{with}\;\log(\sigma_{i}(t_{ij}))=2.4+\tau_{0i}+(0.05+\tau_{1i})\times t_{ij} \end{array}\right.$$

with $u_{i}={\left(b_{0i},b_{1i},\tau_{0i},\tau_{1i}\right)}^{⊤}∼𝒩(0,\sum )$.

Visit times were generated using a uniform distribution around fixed time points (± 1 month) between 0 and 5 years.

Competing event times $T_{ik}^{*}\;(k=1,2)$ were generated using the Brent's univariate root‐finding method [25] according to the following proportional hazards models:

(6) $$\left\{\begin{array}{l} \lambda_{i1}(t)=1.1^{2}t^{1.1^{2}-1}\exp\left(-7+0.02{\tilde{y}}_{i}(t)+0.01{\tilde{y}}_{i}^{\prime }(t)+0.07\sigma_{i}(t)\right)\\ \lambda_{i2}(t)=1.3^{2}t^{1.3^{2}-1}\exp\left(-4-0.01{\tilde{y}}_{i}(t)-0.14{\tilde{y}}_{i}^{\prime }(t)+0.15\sigma_{i}(t)\right) \end{array}\right.$$

Individuals were censored at $C_{i}$ the last visit observed for subject i in the dataset. Finally, the observed time was defined by $T_{i}=\min(T_{i1}^{*},T_{i2}^{*},C_{i})$. Measures of the marker Y posterior to $T_{i}$ were removed from the datasets.

We now describe the four main simulation scenarios considered. Scenario A was performed with a maximum of seven times of measurements, at 0 year, 0.5 year, and then one per year until 5 years. The random effects of the mean $b_{i}$ were supposed to be independent from the random effects of the variance, $\tau_{i}$ using the following matrix of covariance:

$$\begin{array}{ll} \sum & =\left[\begin{array}{cccc} \mathrm{Var}(b_{0i}) & \text{Cov}(b_{0i},b_{1i}) & 0 & 0\\ \text{Cov}(b_{0i},b_{1i}) & \mathrm{Var}(b_{1i}) & 0 & 0\\ 0 & 0 & \mathrm{Var}(\tau_{0i}) & \text{Cov}(\tau_{0i},\tau_{1i})\\ 0 & 0 & \text{Cov}(\tau_{0i},\tau_{1i}) & \mathrm{Var}(\tau_{1i}) \end{array}\right]\\ & =\left[\begin{array}{cccc} 207.36 & -17.4 & 0 & 0\\ -\;17.4 & 9.28 & 0 & 0\\ 0 & 0 & 0.0001 & -0.0006\\ 0 & 0 & -0.0006 & 0.0157 \end{array}\right] \end{array}$$

Scenario B was generated with a maximum of 13 times of measurements, at 0 year, every 3 months the first year and then twice per year until 5 years (as in the PROGRESS trial) and the random effects of the mean $b_{i}$ were also supposed to be independent from the random effects of the variance, $\tau_{i}$. For Scenarios C and D, we have supposed correlated random effects using the following matrix of correlation:

$$\begin{array}{ll} \sum & =\left[\begin{array}{cccc} \mathrm{Var}(b_{0i}) & \text{Cov}(b_{0i},b_{1i}) & \text{Cov}(b_{0i},\tau_{0i}) & \text{Cov}(b_{0i},\tau_{1i})\\ \text{Cov}(b_{0i},b_{1i}) & \mathrm{Var}(b_{1i}) & \text{Cov}(b_{1i},\tau_{0i}) & \text{Cov}(b_{1i},\tau_{1i})\\ \text{Cov}(b_{0i},\tau_{0i}) & \text{Cov}(b_{1i},\tau_{0i}) & \mathrm{Var}(\tau_{0i}) & \text{Cov}(\tau_{0i},\tau_{1i})\\ \text{Cov}(b_{0i},\tau_{1i}) & \text{Cov}(b_{1i},\tau_{1i}) & \text{Cov}(\tau_{0i},\tau_{1i}) & \mathrm{Var}(\tau_{1i}) \end{array}\right]\\ & =\left[\begin{array}{cccc} 210.25 & -15.95 & 2.9 & -0.145\\ -\;15.95 & 9.05 & -0.304 & 0.067\\ 2.9 & -0.304 & 0.1309 & -0.0206\\ -\;0.145 & 0.067 & -0.0206 & 0.0141 \end{array}\right] \end{array}$$

Scenario C was performed with the same visit times as in Scenario A and Scenario D with the same visit times as in Scenario B. Each scenario was considered with N=500 and N=1000.

The simulations were repeated 300 times for each scenario, using S1=500 and S2=5000 draws for the QMC integration approximation.

### 3.3 Estimated Models

To estimate the parameters, we fitted the following model for all scenarios:

(7) $$\left\{\begin{array}{l} Y(t_{ij})=\beta_{0}+b_{0i}+(\beta_{1}+b_{1i})\times t_{ij}+\epsilon_{i}(t_{ij})\\ \epsilon_{i}(t_{ij})∼𝒩(0,\sigma_{i}^{2}(t_{ij}))\;\text{with}\;\log(\sigma_{i}(t_{ij}))=\mu_{0}+\tau_{0i}+(\mu_{1}+\tau_{1i})\times t_{ij}\\ \lambda_{ik}(t)=\lambda_{0k}(t)\exp(\alpha_{1k}{\tilde{y}}_{i}(t)+\alpha_{2k}{\tilde{y}}_{i}^{\prime }(t)+\alpha_{\sigma k}\sigma_{i}(t)) \end{array}\right.$$

with $\lambda_{0k}(t)=\kappa_{k}t^{\kappa_{k}-1}e^{\zeta_{0k}}$. We assumed no constraint for the covariance matrix ∑ for Scenario C and Scenario D, whereas we assumed independence between b and τ for Scenarios A and B.

### 3.4 Performance Measures

For each of the 300 replications, we estimated model parameters, as well as their standard errors and 95% confidence intervals. Using 300 replications, we computed four performance measures: the bias, the asymptotic standard error (ASE), the empirical standard error (ESE), and the empirical coverage (CR) of 95% confidence intervals. Empirical coverage was computed as the proportion of replications of our simulation study in which the estimated 95% confidence interval contained the true parameter value. With 300 replications, the Monte Carlo error for the coverage rate in our simulations was $\sqrt{0.95\times 0.05/300}=1.26\%$, allowing the estimated CR to vary within [92.5,97.5].

### 3.5 Results

Tables 1, 2, 3 report the mean estimates, the bias, the empirical, and mean asymptotic standard errors of the estimated parameters and the coverage rates of their 95% confidence intervals for scenarios A, B, and C on 500 individuals. Results for scenario D and for larger sample size are in the Supporting Information (Tables S1 to S5). The estimation procedure provided satisfactory results for the four scenarios of simulations. Indeed, the bias was minimal, the mean asymptotic standard errors and the empirical standard errors were close, and the coverage rates of the 95% confidence interval were close to the nominal value and most often within the interval [92.5,97.5]. We note that the bias is minimal from the first step, using S1=500 QMC draws, but the second step, using S2=5000 QMC draws, helps to reduce the difference between the mean asymptotic and the empirical standard deviations and thus improve the coverage rates. We observed a slight undercoverage for some parameters of the random‐effects covariance matrix. This small bias mainly affects covariance parameters that are expected to have negligible impact on individual risk prediction. To assess the impact of small biases in the variance parameter estimates on the individual predictions, we computed the individual predictions of the disease risk between years 3 and 5 among subjects event free at 3 years and given measurements collected up to 3 years (using Equation (4)). On a set of 100 datasets generated according to scenario B, Figure S1.A shows that the individual predictions calculated from the estimated parameters are globally equivalent to those obtained using the true parameter values used to generate the data. Moreover, the individual predictions using all estimated parameters are very close to the predictions obtained by replacing the estimates of variance parameters by their true values (see Figure S1.B). Figure S1 confirms the overall quality of the estimated individual prediction and that the small bias on variance parameters has little impact on the individual predictions. Furthermore, on a simulated dataset, we computed also the 95% confidence interval of the predicted probability, using the approach described in Section 2.3 with L=1000. The true prediction probability systematically belongs to the confidence interval (the coverage rate was 100%).

**TABLE 1:** Simulation results for scenario A with 500 subjects (7 measures, $b_{i}$ and $\tau_{i}$ independent).^a^

Parameter		True value	Step 1 (S1=500 QMC)					Step 2 (S2=5000 QMC)				
			Mean	Bias	ESE	ASE	CR (%)	Mean	Bias	ESE	ASE	CR (%)
Longitudinal submodel												
*Intercept*	$\beta_{0}$	142	142.1	0.1	0.736	0.717	94.3	142.1	0.1	0.730	0.728	95.0
*Slope*	$\beta_{1}$	3	2.943	−0.057	0.288	0.253	90.0	2.945	−0.055	0.280	0.271	92.6
*Variability*	$\mu_{0}$	2.4	2.396	−0.004	0.027	0.026	94.7	2.395	−0.005	0.027	0.027	95.3
	$\mu_{1}$	0.05	0.050	0	0.017	0.015	93.0	0.051	0.001	0.016	0.016	94.3
$\sum_{b}$	$\sigma_{b_{0}}^{2}$	207.36	205.0	−2.36	17.31	16.17	91.97	205.1	−2.26	17.19	16.60	91.97
	$\sigma_{b_{0}b_{1}}$	−17.28	−16.06	1.22	4.132	3.663	87.96	−16.04	1.24	3.936	4.020	92.64
	$\sigma_{b_{1}}^{2}$	9.28	9.351	0.071	1.673	1.308	84.95	9.279	−0.001	1.536	1.465	91.64
$\sum_{\tau }$	$\sigma_{\tau_{0}}^{2}$	0.0001	0.004	0.0039	0.006	0.006	98.66	0.004	0.0039	0.007	0.006	98.33
	$\sigma_{\tau_{0}\tau_{1}}$	−0.0006	−0.002	0.0014	0.005	0.005	92.64	−0.003	0.0024	0.005	0.006	95.99
	$\sigma_{\tau_{1}}^{2}$	0.0157	0.016	0.0003	0.005	0.004	91.97	0.017	0.0013	0.005	0.005	93.31
*Survival submodel 1*												
*Current variance*	$\alpha_{\sigma 1}$	0.07	0.064	−0.006	0.041	0.039	94.7	0.064	−0.006	0.041	0.039	95.3
*Current value*	$\alpha_{11}$	0.02	0.020	0	0.008	0.007	95.7	0.020	0	0.008	0.007	95.3
*Current slope*	$\alpha_{21}$	0.01	0.008	−0.002	0.072	0.066	94.3	0.007	−0.003	0.071	0.067	96.0
*Weibull*	$\sqrt{\kappa_{1}}$	1.1	1.099	−0.001	0.056	0.059	97.7	1.098	−0.002	0.056	0.059	98.0
	$\zeta_{01}$	−7	−6.884	0.116	1.257	1.199	94.3	−6.885	0.115	1.257	1.204	94.7
*Survival submodel 2*												
*Current variance*	$\alpha_{\sigma 2}$	0.15	0.169	0.019	0.091	0.046	92.3	0.168	0.018	0.091	0.054	96.3
*Current value*	$\alpha_{12}$	−0.01	−0.012	−0.002	0.014	0.009	96.3	−0.012	−0.002	0.015	0.010	96.7
*Current slope*	$\alpha_{22}$	−0.14	−0.171	0.031	0.173	0.086	92.6	−0.167	0.027	0.170	0.095	94.7
*Weibull*	$\sqrt{\kappa_{2}}$	1.3	1.310	0.010	0.102	0.079	97.0	1.311	0.011	0.105	0.084	98.0
	$\zeta_{02}$	−4	−4.075	−0.075	1.389	1.366	96.0	−4.075	−0.075	1.388	1.401	95.7

Abbreviations: ASE: asymptotic standard error; coverage rate: coverage rate of the 95% confidence interval; ESE: empirical standard error.

^a^ Results for 299 replicates with complete convergence over 300.

**TABLE 2:** Simulation results for scenario B with 500 subjects (13 measures, $b_{i}$ and $\tau_{i}$ independent).^a^

Parameter		True value	Step 1 (S1=500 QMC)					Step 2 (S2=5000 QMC)				
			Mean	Bias	ESE	ASE	CR (%)	Mean	Bias	ESE	ASE	CR (%)
Longitudinal submodel												
*Intercept*	$\beta_{0}$	142	142.0	0	0.779	0.655	90.7	142.0	0	0.767	0.721	92.7
*Slope*	$\beta_{1}$	3	2.996	−0.004	0.252	0.201	90.0	3.000	0	0.248	0.235	93.3
*Variability*	$\mu_{0}$	2.4	2.402	0.002	0.019	0.018	92.0	2.401	0.001	0.019	0.018	92.7
	$\mu_{1}$	0.05	0.047	−0.003	0.012	0.011	92.0	0.049	−0.001	0.012	0.012	94.0
$\sum_{b}$	$\sigma_{b_{0}}^{2}$	207.36	208.1	0.74	17.79	14.18	87.00	208.0	0.64	17.29	15.92	92.33
	$\sigma_{b_{0}b_{1}}$	−17.28	−15.74	1.54	4.165	2.954	77.00	−15.85	1.43	4.022	3.614	85.00
	$\sigma_{b_{1}}^{2}$	9.28	9.236	−0.044	1.476	0.948	79.00	9.256	0.024	1.322	1.246	89.67
$\sum_{\tau }$	$\sigma_{\tau_{0}}^{2}$	0.0001	0.002	0.0019	0.003	0.002	97.33	0.002	0.0019	0.003	0.003	97.67
	$\sigma_{\tau_{0}\tau_{1}}$	−0.0006	−0.001	−0.0004	0.003	0.003	91.33	−0.001	−0.0004	0.003	0.004	93.67
	$\sigma_{\tau_{1}}^{2}$	0.0157	0.015	−0.0007	0.003	0.003	88.67	0.016	0.0003	0.003	0.003	94.67
*Survival submodel 1*												
*Current variance*	$\alpha_{\sigma 1}$	0.07	0.063	−0.007	0.030	0.028	92.7	0.065	−0.005	0.029	0.028	93.7
*Current value*	$\alpha_{11}$	0.02	0.020	0	0.006	0.007	96.3	0.020	0	0.006	0.007	96.3
*Current slope*	$\alpha_{21}$	0.01	0.007	−0.003	0.057	0.053	96.3	0.007	−0.003	0.055	0.055	96.0
*Weibull*	$\sqrt{\kappa_{1}}$	1.1	1.106	0.006	0.055	0.054	94.0	1.105	0.005	0.055	0.055	95.0
	$\zeta_{01}$	−7	−6.912	0.088	0.992	1.042	95.7	−6.914	0.086	0.992	1.050	95.7
*Survival submodel 2*												
*Current variance*	$\alpha_{\sigma 2}$	0.15	0.158	0.008	0.034	0.030	92.7	0.159	0.009	0.031	0.032	97.3
*Current value*	$\alpha_{12}$	−0.01	−0.010	0	0.008	0.008	94.7	−0.010	0	0.008	0.008	95.3
*Current slope*	$\alpha_{22}$	−0.14	−0.146	−0.006	0.064	0.061	94.0	−0.145	−0.005	0.063	0.064	95.0
*Weibull*	$\sqrt{\kappa_{2}}$	1.3	1.299	−0.001	0.067	0.067	95.7	1.299	−0.001	0.066	0.068	96.3
	$\zeta_{02}$	−4	−4.104	−0.104	1.173	1.111	93.7	−4.107	−0.107	1.173	1.139	93.7

Abbreviations: ASE: asymptotic standard error; coverage rate: coverage rate of the 95% confidence interval; ESE: empirical standard error.

^a^ Results for 300 replicates with complete convergence over 300.

**TABLE 3:** Simulation results for Scenario C with 500 subjects (7 measures, $b_{i}$ and $\tau_{i}$ correlated).^a^

Parameter		True value	Step 1 (S1=500 QMC)					Step 2 (S2=5000 QMC)				
			Mean	Bias	ESE	ASE	CR (%)	Mean	Bias	ESE	ASE	CR (%)
Longitudinal submodel												
*Intercept*	$\beta_{0}$	142	141.9	−0.001	0.833	0.742	92.3	141.9	−0.001	0.820	0.756	93.3
*Slope*	$\beta_{1}$	3	3.019	0.019	0.320	0.282	90.6	3.023	0.023	0.314	0.290	92.0
*Variability*	$\mu_{0}$	2.4	2.401	0.001	0.035	0.033	93.6	2.399	−0.001	0.035	0.033	94.3
	$\mu_{1}$	0.05	0.050	0	0.016	0.015	92.6	0.050	0	0.016	0.016	93.7
$\sum_{b\tau }$	$\sigma_{b_{0}}^{2}$	210.25	209.7	−0.55	20.69	16.90	88.23	209.4	−0.85	20.39	17.72	91.33
	$\sigma_{b_{0}b_{1}}$	−15.95	−15.43	0.52	4.734	3.942	88.59	−15.48	0.47	4.398	4.209	92.33
	$\sigma_{b_{0}\tau_{0}}$	2.9	2.796	−0.104	0.610	0.521	88.93	2.812	−0.088	0.592	0.533	89.67
	$\sigma_{b_{0}\tau_{1}}$	−0.145	−0.129	0.016	0.251	0.210	89.54	−0.115	0.03	0.240	0.220	93.00
	$\sigma_{b_{1}}^{2}$	9.05	9.181	0.131	1.745	1.371	85.91	9.084	0.034	1.599	1.479	91.33
	$\sigma_{b_{1}\tau_{0}}$	−0.304	−0.295	0.009	0.178	0.157	91.95	−0.291	0.013	0.169	0.162	94.67
	$\sigma_{b_{1}\tau_{1}}$	0.067	0.069	0.002	0.073	0.061	88.93	0.065	−0.002	0.068	0.063	92.00
	$\sigma_{\tau_{0}}^{2}$	0.1309	0.123	−0.0079	0.029	0.026	87.53	0.128	−0.0029	0.029	0.028	91.67
	$\sigma_{\tau_{0}\tau_{1}}$	−0.0206	−0.019	0.0016	0.011	0.008	87.53	−0.021	−0.0004	0.010	0.010	94.00
	$\sigma_{\tau_{1}}^{2}$	0.0141	0.014	−0.0001	0.005	0.004	80.87	0.015	0.0009	0.005	0.005	94.33
*Survival submodel 1*												
*Current variance*	$\alpha_{\sigma 1}$	0.07	0.065	−0.005	0.051	0.046	93.3	0.067	−0.003	0.050	0.047	94.7
*Current value*	$\alpha_{11}$	0.02	0.021	0.001	0.012	0.011	93.3	0.021	0.001	0.012	0.011	93.3
*Current slope*	$\alpha_{21}$	0.01	−0.004	−0.014	0.077	0.076	93.6	−0.051	−0.061	0.817	0.470	94.0
*Weibull*	$\sqrt{\kappa_{1}}$	1.1	1.094	−0.006	0.052	0.054	96.3	1.095	−0.005	0.052	0.055	96.7
	$\zeta_{01}$	−7	−7.133	−0.133	1.437	1.296	94.0	−6.958	0.042	3.204	2.612	94.7
*Survival submodel 2*												
*Current variance*	$\alpha_{\sigma 2}$	0.15	0.166	0.016	0.079	0.051	91.3	0.165	0.015	0.077	0.057	95.0
*Current value*	$\alpha_{12}$	−0.01	−0.016	−0.006	0.018	0.013	92.0	−0.012	−0.002	0.017	0.014	93.7
*Current slope*	$\alpha_{22}$	−0.14	−0.154	−0.014	0.113	0.088	95.6	−0.178	−0.038	0.486	0.253	95.7
*Weibull*	$\sqrt{\kappa_{2}}$	1.3	1.314	0.014	0.078	0.069	94.3	1.314	0.014	0.074	0.072	94.7
	$\zeta_{02}$	−4	−4.084	−0.084	1.758	1.524	94.3	−3.983	0.017	2.469	2.109	95.0

Abbreviations: ASE: asymptotic standard error; coverage rate: coverage rate of the 95% confidence interval; ESE: empirical standard error.

^a^ Results for 298 replicates with complete convergence over 300 for step 1 and for 300 replicates with complete convergence over 300 for step 2.

This simulation study also illustrates the impact of the choice of the number of QMC draws S1 and S2 on the computation time: for scenario A, the medians of computation time are around 13 min (25 iterations in median) and 9 min (1 iteration in median), respectively, for steps 1 and 2, on 10 cores.

### 3.6 Additional Simulation Scenarios

Two additional misspecified scenarios (E and F) were performed to assess the robustness of the estimates. In these scenarios, only one event was considered. In Scenario E, the marker was generated with a quadratic time trend but estimated with a linear trend. This scenario was performed to assess the effect of a misspecification of the blood pressure time trend on the estimation of the variance effect on the event risk. As expected, the estimates of the fixed effects in the mixed model are biased, but the estimates of the model for the residual variance and of the time‐to‐event model are robust (Table S6).

In Scenario F, the variance of the marker was generated as constant with time, so without fixed and random slopes, but the estimated model assumed a linear time trend for the variance with fixed and random slopes. This scenario was considered to assess if the estimation algorithm was robust enough to estimate the location‐scale joint model with time‐varying variance when the variance in the data is constant. The estimates remain unbiased with nominal coverage rates. As expected, the estimates of the fixed slope and the variance of the random slope for the residual variance are close to zero (Table S7). Finally, this scenario was also estimated considering a constant variance over time, matching the underlying data generation model. The results showed that the estimates were unbiased, and the coverage rates were consistent with nominal value (Table S8).

## 4 Application

### 4.1 Progress Clinical Trial

We applied the proposed methodology on the data from the PROGRESS clinical trial [16] designed to evaluate a blood‐pressure lowering treatment in secondary prevention. PROGRESS is a multicenter, double‐blind randomized clinical trial including patients with a history of stroke or transient ischemic attack within 5 years before inclusion. Patients were recruited between May 1995 and November 1997. The follow‐up comprised five visits in the first year, then two visits each years until the end of the study or the occurrence of a major CVD (stroke, myocardial infarction, and cerebral hemorrhage) or death. At each visit, blood pressure was measured twice and, as recommended [26], we analyzed the mean of the two measurements at each time. Prior to randomization, eligible patients were subjected to a 4‐week run‐in phase to test their tolerance to the treatment. At randomization, patients assigned to the control group stopped the treatment. In order to avoid an effect of the change of therapy at randomization, we removed the blood pressure measure at randomization. The model was estimated only on Non Asian subjects due to differences between Asians and non Asians with regard to CVD risk and risk factors and because the treatment was not exactly the same. Finally, the current study was conducted over 3710 Non Asian patients, 1856 for the controlled group and 1854 for the treatment group, and included 672 CVD and 150 deaths without CVD. There are 2525 (68%) men and 1185 (32%) women. The average age at entry in the study is 67 years old (sd = 9.8) with a minimum at 26 and a maximum at 91 years old.

### 4.2 Specification of the Model

This study aimed to evaluate the impact of the blood pressure variability on the risk of CVD with or without death and death due to other causes. To do so, we estimated the proposed joint model (Model CVCS+V, for current value, current slope and variance) with heterogeneous time‐dependent variance defined by equations (1) and (2) using the time since the first blood pressure measurement post‐randomization. The trajectory of blood pressure was described over time by a linear mixed‐effect model. The individual time trend of the marker was modeled by a quadratic trend and the residual variance by a linear trend. The baseline hazard functions of both events were defined by B‐splines with three interior knots located at the quantiles of the observed event times. According to the AIC, the model with three knots was better than models with 1 or 5 knots for each baseline hazard function (respectively 298651.9, 298659.8, and 298661.2). The model allowed the risk of each event to depend on the time‐dependent intra‐subject variability, the individual current value and the current slope. The longitudinal submodel and the variance submodel were adjusted for treatment group and survival submodels were adjusted for treatment group, age at baseline, sex (male versus female), and the baseline measurement of blood pressure (BP0) measured before the 4‐week run‐in phase, to reflect the historical blood pressure level:

$$\left\{\begin{array}{l} y(t_{ij})=\beta_{0}+b_{0i}+(\beta_{1}+b_{1i})t_{ij}+(\beta_{2}+b_{2i})t_{ij}^{2}+\beta_{3}trt_{i}\\ \;+\beta_{4}trt_{i}t_{ij}+\beta_{5}trt_{i}t_{ij}^{2}+\epsilon_{i}(t_{ij})\\ \epsilon_{i}(t_{ij})∼N(0,\sigma_{i}^{2}(t_{ij}))\\ \text{with}\;\log(\sigma_{i}(t_{ij}))=\mu_{0}+\tau_{0i}+(\mu_{1}+\tau_{1i})t_{ij}+\mu_{2}trt_{i}\\ \lambda_{ik}(t)=\lambda_{0k}(t)\exp(\gamma_{0k}trt_{i}+\gamma_{1k}male_{i}+\gamma_{2k}age_{i}+\gamma_{3k}BP0_{i}\\ \;+\alpha_{1k}{\tilde{y}}_{i}(t)+\alpha_{2k}{\tilde{y}}_{i}^{\prime }(t)+\alpha_{\sigma k}\sigma_{i}(t)) \end{array}\right.$$

The estimation was performed with S1=500 and S2=10000 draws of QMC to ensure a greater accuracy.

This model was compared with two classical joint model without heterogeneous variance, that is, $\sigma_{i}^{2}(t_{ij})=\sigma^{2}$ for all i=1,…,N and $j=1,\ldots ,n_{i}$. The first one allowed the risk of each event to depend only on the individual current value (CV model) and the second one on both the individual current value and current slope (CVCS model). It was also compared to a location‐scale joint model with a structure similar to that proposed by [15] and [14], featuring time‐fixed (i.e., constant) subject‐specific residual variance and event risks dependent on the random intercept, the random slope and the subject‐specific residual variance.

### 4.3 Results

The AIC from the complete model (298651.9) was clearly better than the AIC from the two joint models with a constant residual variance and a dependence either on the current value only (CV model: 302062.4) or on both the current value and the current slope (CVCS model: 301662.9) and was also better than the joint model assuming a time‐fixed subject‐specific residual variance (298872.2). This highlights the importance of taking into account a time‐dependent subject‐specific variance.

Table 4 provides estimates from the complete joint model and Table 5 presents the covariance matrix of the random effects and their standard errors computed through the Delta‐Method. Blood pressure was lower for individuals from the treatment group ($\beta_{3}=-9.354$, p<0.001). The interactions between randomization and time are not statistically significant; however, when compared to a model without these interaction terms, the full model provides a better fit according to the likelihood ratio test. The variance of the residual error was heterogeneous between the subjects ($\hat{\mathrm{Var}}(\tau_{0i})=0.13$, se=7e−3) and was lower for treated patients ($\hat{\mu_{2}}=-0.033$, p=0.018). The risk of CVD events increased with age (HR=1.04 for one year, IC=[1.03;1.05]) and was higher for men (HR=1.34, IC=[1.13;1.60]). It was also higher for elevated blood pressure values prior to entering the study (HR = 1.03 per 5 mmHg, IC=[1.00;1.06]). Adjusting for age, sex, treatment group, and blood pressure before inclusion, the risk of CVD increased with the current blood pressure variance (HR=1.04, IC=[1.01;1.08]). More surprisingly, the risk decreased when the current value of blood pressure increased (HR=0.93, IC=[0.89;0.98] for 5 mmHg) or when the current slope of blood pressure increased (HR=0.91, IC=[0.87;0.95]). These negative associations are not due to the adjustment for blood pressure variance, as the same results are observed in the CVCS model (see Table S7). The risk of death from other causes was higher for older individuals (HR=1.05, IC=[1.03;1.07] for one year) and for men (HR=1.68, IC=[1.15;2.46]), but it was not associated with the treatment group or the first measurement of blood pressure. More importantly, the death risk significantly increased with the current variance of blood pressure (HR=1.11,IC=[1.03;1.19]) and decreased when the current value (HR=0.90,IC=[0.808;1.002] for 5 mmHg) and the current slope of blood pressure increased (HR=0.89,IC=[0.789;1.002]). As for CVD, these unexpected results regarding the current value and the current slope are also observed without adjustment on the current blood pressure variance (CVCS model in Table S7). However, these results must be interpreted cautiously considering that the study population consists only of subjects who have survived a stroke and are included in a clinical trial, thus benefiting from close monitoring.

**TABLE 4:** Parameter estimates of the joint model on the progress clinical trial data (CVCS+V model).

Parameter	Estimate	Standard error	p
*Survival submodel for CVD*			
BP current variance	0.042	0.017	0.012
BP current value	−0.0145	0.005	0.002
BP current slope	−0.093	0.021	<0.001
BP0	0.006	0.003	0.016
Treatment group	−0.222	0.087	0.011
Male	0.293	0.089	0.001
Age	0.041	0.005	<0.001
*Survival submodel for Death*			
BP current variance	0.102	0.035	0.004
BP current value	−0.021	0.011	0.055
BP current slope	−0.118	0.061	0.051
BP0	−0.002	0.005	0.672
Treatment group	−0.070	0.182	0.700
Male	0.521	0.194	0.007
Age	0.053	0.010	<0.001
*Longitudinal submodel*			
*Blood Pressure Mean*			
Intercept	143.3	0.368	<0.001
Time	−0.108	0.266	0.684
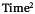	−0.111	0.064	0.084
Treatment group	−9.354	0.518	<0.001
Time × treatment group	0.678	0.364	0.063
Time× treatment 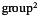	0.005	0.087	0.951
*Blood Pressure Residual Variance*			
Intercept	2.321	0.012	<0.001
Time	0.007	0.004	0.088
Treatment group	−0.033	0.014	0.018

Abbreviation: BP: Blood pressure.

**TABLE 5:** Covariance matrix (and standard errors) of the random effects computed using the Delta‐Method.

$\sum =\left[\begin{array}{ccccc} Var(b_{0i}) & & & & \\ Cov(b_{0i},b_{1i}) & Var(b_{1i}) & & & \\ Cov(b_{0i},b_{2i}) & Cov(b_{1i},b_{2i}) & Var(b_{2i}) & & \\ Cov(b_{0i},\tau_{0i}) & Cov(b_{1i},\tau_{0i}) & Cov(b_{2i},\tau_{0i}) & Var(\tau_{0i}) & \\ Cov(b_{0i},\tau_{1i}) & Cov(b_{1i},\tau_{1i}) & Cov(b_{2i},\tau_{1i}) & Cov(\tau_{0i},\tau_{1i}) & Var(\tau_{1i}) \end{array}\right]=\left[\begin{array}{ccccc} 214.4_{\left(6.1\right)} & & & & \\ -\;25.3_{\left(3.2\right)} & 41.5_{\left(3.1\right)} & & & \\ 0.95_{\left(0.71\right)} & -7.4_{\left(0.69\right)} & 1.7_{\left(0.2\right)} & & \\ 2.2_{\left(0.2\right)} & -0.14_{\left(0.10\right)} & -0.04_{\left(0.02\right)} & 0.13_{\left(7e-3\right)} & \\ -\;0.24_{\left(0.07\right)} & 0.25_{\left(0.05\right)} & -0.02_{\left(0.01\right)} & -0.02_{\left(2e-3\right)} & 0.01_{\left(1e-3\right)} \end{array}\right]$

### 4.4 Goodness‐of‐Fit Assessment

To assess the fit of the time‐to‐event submodels, we computed for each event, the predicted cumulative hazard function at each event time by plugging the empirical Bayes estimates of the random effects in the formula for the risk function. Then we compared the mean of this predicted cumulative hazard function with its Nelson–Aalen estimator for the whole sample (Figure S2) and stratified according to sex and randomization group (Figure S3). These figures show that the joint model adequately fitted both risks and that the proportional risk assumption was valid for each categorical variable.

To evaluate the fit of the longitudinal submodel, we computed the empirical Bayes estimates of the random effects and the predicted marker values for each individual at their respective visit times. Figure S4 compares the mean predicted marker values at each visit with the mean of the observed measurements, demonstrating that the joint model accurately captures the trajectory of blood pressure.

To highlight the impact of adding a subject‐specific and time‐dependent residual variance in the mixed model, we computed the individual predictions of the marker over time for some selected subjects. The predicted value of blood pressure corresponds to the conditional expectation given the random effects, defined by $\hat{𝔼}(Y_{i}(t)|{\tilde{b}}_{i},{\tilde{\tau }}_{i})$ and the prediction interval around these predicted values is given by $\hat{𝔼}(Y_{i}(t)|{\tilde{b}}_{i},{\tilde{\tau }}_{i})\pm 1.96\hat{𝕍}(Y_{i}(t)|{\tilde{b}}_{i},{\tilde{\tau }}_{i})$. For each subject, the empirical Bayes estimates of the random effects, denoted by $\left({\tilde{b}}_{i},{\tilde{\tau }}_{i})=\text{argmax}f(b_{i},\tau_{i}|Y_{i},T_{i},\delta_{i}\right)$, corresponds to the mode of their estimated conditional posterior given the data. They are computed by maximising $f(Y_{i},T_{i},\delta_{i})f(b_{i},\tau_{i})$ with the Marquardt–Levenberg algorithm.

**FIGURE 1:**
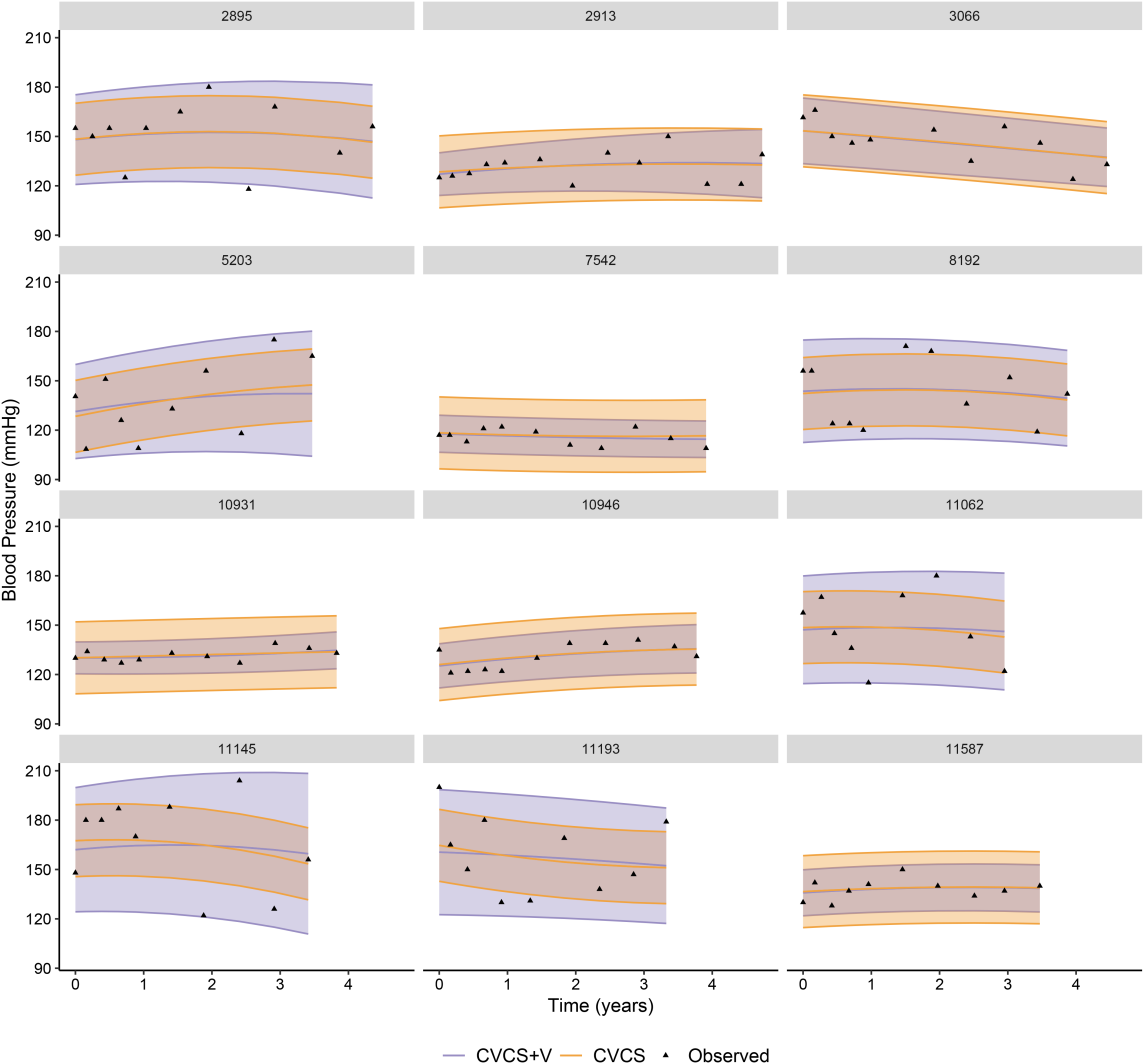
Prediction over time of the individual blood pressure and its prediction interval at 95% for 12 subjects. Model CVCS+V assumed a time‐dependent subject‐specific variability and the model CVCS assumes a homogeneous and constant variability. The black triangles are the observed measurements.

For some selected subjects still at risk at three years, Figure 1 presents the predicted values and their confidence intervals from the models with and without subject‐specific residual variance (CVCS+V and CVCS). It shows that assuming a time‐dependent and subject‐specific residual variance allows a better fit of the uncertainty around the individual prediction.

### 4.5 Risk Predictions

We compared the predictive abilities of models with and without time‐dependent individual variability using AUC using a fivefold cross‐validation. The individual predictions of having CVD (or death from other causes) between three and five years for subjects free of any event at three years were computed using equation (4). The AUC was computed using the timeROC package [27]. The results are slightly better for the model with heterogeneous variability. We obtained, respectively, 0.559 (0.064) and 0.537 (0.064) for the risk of CVD and 0.624 (0.080) and 0.625 (0.080) for the risk of death. Note that between three and five years, only 52 deaths were observed, compared to 417 CVD during the same period.

**FIGURE 2:**
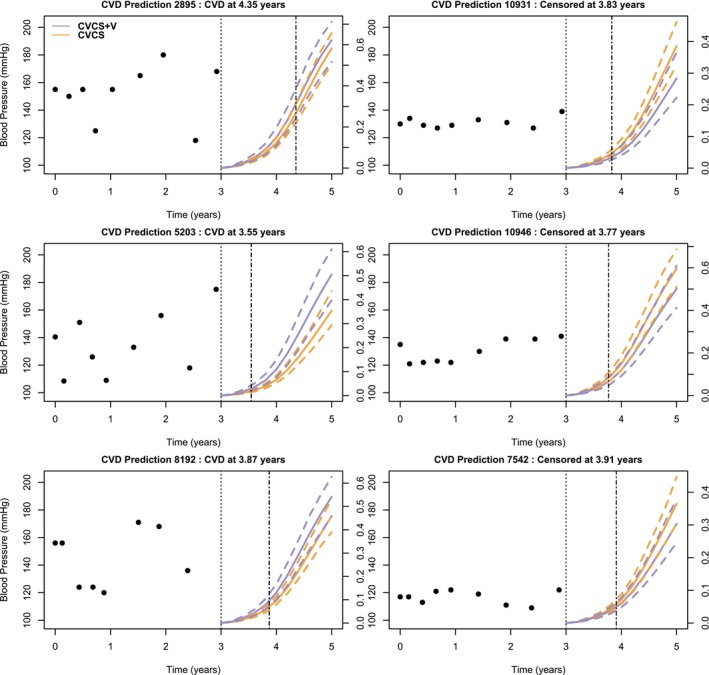
Prediction of the risk of CVD between three and five years (with its 95% confidence interval indicated by dashed lines), for six patients at risk at three years, for Model CVCS+V (purple) and Model CVCS (orange). The dashed lines represent the observed time.

**FIGURE 3:**
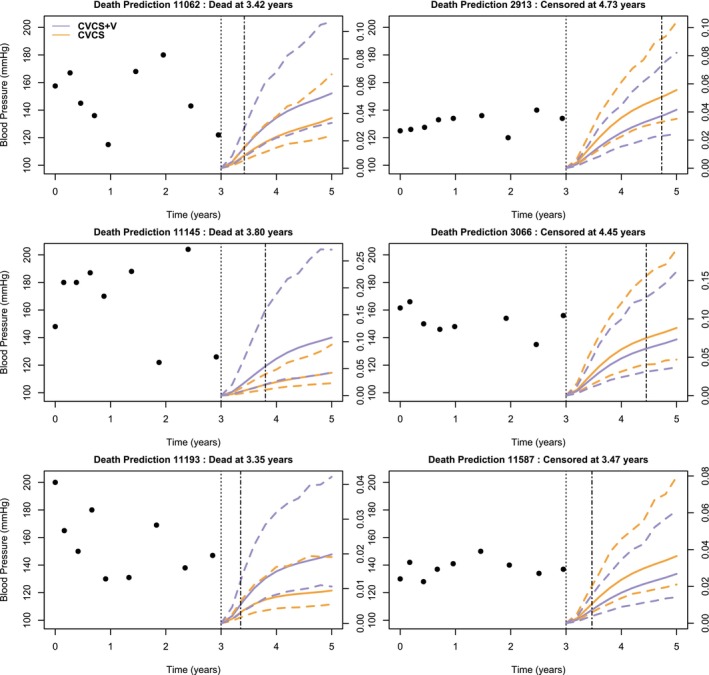
Prediction of the risk of Death between three and five years (with its 95% confidence interval indicated by dashed lines), for six patients at risk at three years, for Model CVCS+V (purple) and Model CVCS (orange). The dashed lines represent the observed time.

To illustrate the effect of taking into account the current value of individual variance, we also computed the predicted risk of the events between three and five years for different subjects from both models, with and without time‐dependent individual variability. We used the subjects selected for Figure 1 and present their predictions obtained via the cross‐validation procedure. Figures 2 and 3 show that, for both the risk of CVD and the risk of death, the predicted probability is higher with the complete model when the individual experienced the event between three and five years than with the model without the heterogeneous variability. Conversely, the predicted risk is smaller with the complete model when the individual do not experience the corresponding event.

## 5 Discussion

In this work, we have proposed a new joint model with a subject‐specific time‐dependent variance that extends the models proposed by Gao et al. [14] and Barrett et al. [15]. Indeed, this new model allows time and covariate dependent individual variance and a flexible dependence structure between the competing events and the longitudinal marker. In particular, the risk of events may depend on both the current value and the current slope of the marker, in addition to the subject‐specific time‐dependent standard deviation of the residual error. This is an important asset of the model given that, in most health research contexts, it is more sensible to assume that the event risk depends on the time‐dependent current value or slope of the marker instead of only time‐independent random effects. Moreover, accounting for competing events may be important in many clinical applications. Simulation study allows us to demonstrate the good performance of the estimation procedure and to study the impact of the choice of the number of QMC draws for numerical integration. The model converged without bias and with good coverage rates. Moreover, the estimates of the time‐to‐event sub‐model are quite robust to a misspecification of the marker trajectory. In addition, we provided an R‐package that allows frequentist estimation with a robust estimation algorithm which had shown very good behaviour in our simulations and in a previous work with different models [20].

The analysis of the PROGRESS trial has shown that a high variability of blood pressure is associated with a high risk of CVD and death from other causes. Moreover, the individual residual variability depends on treatment group. The counterintuitive findings regarding the effects of current blood pressure value and slope are not attributable to the correlation between individual slope and individual variance. In fact, similar results were observed in the standard joint model, which does not account for subject‐specific variance. Additionally, the studied population is highly specific‐consisting of individuals with a history of stroke and under tight clinical control‐limiting the generalizability of these findings to other groups. Replicating this analysis in representative samples of the general population would be highly valuable for determining whether blood pressure variability could be a target for primary prevention of cardiovascular disease, mortality, or stroke.

The proposed model is flexible enough to accommodate nonlinear time trends for the marker, including polynomial forms or smooth functions based on a spline basis. Additionally, it can handle time‐varying, subject‐specific residual variance but can also be applied under the assumption of constant subject‐specific residual variance. We have not considered more flexible time trend for the variance in the application because, as in most data sets, the number of repeated measurements in the PROGRESS trial is not large enough for estimating such a flexible model. However, the use of more flexible time trend is possible in the package.

In this work, we have supposed that the visit times were not informative and that missing measurements before the event were missing at random. In the PROGRESS clinical study, these hypotheses are quite plausible since visits were planed following a prespecified protocol and the rate of missed visits before the event was low (less than 3%). For application to observational studies, it could be useful to extend this approach to consider an informative observation process. However, such a model would require three submodels: A mixed model for the evolution of the marker, a submodel for repeated events to describe the visit process and a model for the competing events of interest. This model would rely on non‐verifiable parametric assumptions and its estimation process would be much more cumbersome.

The proposed approach addressed both right censoring and left truncation, the two most common observation schemes for time‐to‐event data. Considering interval censoring and semi‐competing events could represent a valuable enhancement. This extension would be useful when the exact time of onset of the main event is unknown (dementia for instance) and the competing event may arise after the main event (death). However, this would require modeling the three transition intensities and the interval censoring would significantly complicate the computation of the likelihood.

Such joint models with dependence on the heterogeneous variance (that can be viewed as an extension of the location‐scale mixed model [13]) are of great interest to investigate the association between the variability of markers or risk factors and the risk of health events in various fields of medical research, possibly allowing to improve the prediction ability for the event. For instance, hypotheses have emerged about the link between emotional instability and the risk of psychiatric events, or the variability of glycemia and the prognosis of diabetes. Thanks to wearable devices, recent medical research studies often include frequent repeated measures of exposures or biomarkers, allowing the investigation of hypotheses regarding the variability.

## Author Contributions

Léonie Courcoul contributed to the definition of the model and design of the simulation, implemented all the codes, performed the simulation study and the real data analysis, and wrote the first draft of the manuscript. Hélène Jacqmin‐Gadda and Antoine Barbieri supervised the work (model definition and implementation, simulation design and real data analysis), contributed to the interpretation of results and revised the manuscript. Christophe Tzourio and Mark Woodward made the data available, contributed to the design and interpretation of the real data analysis and revised the manuscript.

## Conflicts of Interest

The authors declare no conflicts of interest.

## Supporting information




**Data S1.** Supporting Information.

## Data Availability

Data may be available from contacting Professor Woodward markw@georgeinstitute.org.au, subject to legal considerations.
