# Supplementary material for: A Location‐Scale Joint Model for Studying the Link Between the Time‐Dependent Subject‐Specific Variability of Blood Pressure and Competing Events
Source: Stat Med. 2025 Sep 5;44(20-22):e70244. doi: 10.1002/sim.70244 (PMC12412725; doi:10.1002/sim.70244)
Supplement: Supplementary file 1 — Data S1. Supporting Information. [file SIM-44-0-s001.pdf]

A location-scale joint model for studying the link between the time-dependent subject-specific variability of blood pressure and competing events

Léonie Courcoul[1*], Christophe Tzourio[1], Mark Woodward[2,3], Antoine Barbieri[1], and Hélène Jacqmin-Gadda[1]

[1]Univ. Bordeaux, INSERM, Bordeaux Population Health, U1219, France
[2] The George Institute for Global Health, Imperial College London, UK
[3] The George Institute for Global Health, University of New South Wales, Sydney, Australia

TABLE S1 – Simulation results for scenario A with 1000 subjects (7 measures, b_i and τ_i independent).*

**Parameter**			**Step 1 (S1=500 QMC)**					**Step 2 (S2=5000 QMC)**				
		**True value**	**Mean**	**Bias**	**ESE**	**ASE**	**CR (%)**	**Mean**	**Bias**	**ESE**	**ASE**	**CR (%)**
Longitudinal submodel												
Intercept	β_0	142	142.0	0	0.533	0.509	94.97	142.0	0	0.530	0.515	95.33
Slope	β_1	3	2.987	-0.013	0.189	0.181	94.63	2.985	-0.015	0.184	0.194	96.33
Variability	μ_0	2.4	2.399	-0.001	0.019	0.019	94.30	2.398	-0.002	0.019	0.019	94.00
	μ_1	0.05	0.050	0	0.012	0.011	91.95	0.050	0	0.012	0.012	94.67
Σ_b	$\sigma^2_{b_0}$	207.36	205.9	-1.46	11.68	11.11	94.30	205.9	-1.46	11.67	11.74	95.67
	$\sigma_{b_0 b_1}$	-17.28	-15.70	1.58	3.089	2.658	86.91	-15.76	1.52	2.947	2.893	90.33
	$\sigma^2_{b_1}$	9.28	9.096	-0.184	1.183	0.935	85.91	9.096	-0.184	1.118	1.058	92.00
Σ_τ	$\sigma^2_{\tau_0}$	0.0001	0.002	0.0019	0.004	0.003	99.33	0.003	0.0029	0.005	0.004	97.67
	$\sigma_{\tau_0 \tau_1}$	-0.0006	-0.002	-0.0014	0.003	0.003	95.64	-0.002	-0.0014	0.004	0.004	95.33
	$\sigma^2_{\tau_1}$	0.0157	0.016	0.0003	0.003	0.003	92.62	0.017	0.0013	0.004	0.004	95.00
Survival submodel 1												
Current variance	$\alpha_{\sigma 1}$	0.07	0.066	-0.004	0.028	0.026	94.63	0.067	-0.003	0.027	0.026	94.67
Current value	α_{11}	0.02	0.020	0	0.005	0.005	94.63	0.020	0	0.006	0.005	94.33
Current slope	α_{21}	0.01	0.012	0.002	0.045	0.046	95.64	0.011	0.001	0.045	0.047	95.00
Weibull	$\sqrt{\kappa_1}$	1.1	1.094	-0.006	0.042	0.041	93.96	1.094	-0.006	0.042	0.041	94.33
	ζ_{01}	-7	-6.991	0.009	0.837	0.820	95.30	-6.990	0.010	0.838	0.825	95.00
Survival submodel 2												
Current variance	$\alpha_{\sigma 2}$	0.15	0.155	0.005	0.034	0.029	90.94	0.155	0.005	0.033	0.032	94.33
Current value	α_{12}	-0.01	-0.010	0	0.006	0.006	94.30	-0.010	0	0.006	0.006	94.33
Current slope	α_{22}	-0.14	-0.143	-0.003	0.060	0.054	95.97	-0.143	-0.003	0.058	0.057	96.67
Weibull	$\sqrt{\kappa_2}$	1.3	1.297	-0.003	0.054	0.053	94.97	1.297	-0.003	0.054	0.055	96.00
	ζ_{02}	-4	-4.035	-0.035	0.919	0.897	96.31	-4.035	-0.035	0.915	0.916	97.67

ESE : Empirical Standard Error ; ASE : Asymptotic Standard Error ; Coverage rate : coverage rate of the 95% confidence interval.

* Results for 298 replicates with complete convergence over 300 for step 1 and 300 replicates over 300 for step 2.

Table S2 – Simulation results for scenario B with 1000 subjects (13 measures, b_i and τ_i independent).*

**Parameter**			**Step 1 (S1 = 500 QMC)**					**Step 2 (S2 = 5000 QMC)**				
		**True value**	**Mean**	**Bias**	**ESE**	**ASE**	**Coverage (%)**	**Mean**	**Bias**	**ESE**	**ASE**	**Coverage (%)**
Longitudinal submodel												
Intercept	β_0	142	142.0	0	0.544	0.476	91.30	142.0	0	0.539	0.500	93.33
Slope	β_1	3	2.993	-0.007	0.184	0.149	89.97	2.992	-0.008	0.174	0.162	93.33
Variability	μ_0	2.4	2.401	0.001	0.012	0.013	94.98	2.400	0	0.012	0.013	95.33
	μ_1	0.05	0.048	-0.002	0.008	0.008	92.31	0.049	-0.001	0.008	0.008	93.33
Σ_b	$\sigma^2_{b_0}$	207.36	209.9	2.5	12.91	10.44	88.63	209.6	2.2	12.61	11.03	90.00
	$\sigma_{b_0 b_1}$	-17.28	-15.75	1.53	3.140	2.182	78.60	-15.87	1.41	2.915	2.433	84.33
	$\sigma^2_{b_1}$	9.28	9.161	-0.119	1.083	0.724	78.60	9.147	-0.133	0.999	0.855	87.67
Σ_τ	$\sigma^2_{\tau_0}$	0.0001	0.001	0.0009	0.002	0.001	93.65	0.001	0.0009	0.003	0.002	94.00
	$\sigma_{\tau_0 \tau_1}$	-0.0006	-0.001	-0.0004	0.002	0.002	92.98	-0.001	-0.0004	0.002	0.002	94.00
	$\sigma^2_{\tau_1}$	0.0157	0.015	-0.0007	0.002	0.002	89.97	0.016	0.0003	0.002	0.0023	93.33
Survival submodel 1												
Current variance	$\alpha_{\sigma 1}$	0.07	0.063	-0.007	0.020	0.019	92.31	0.064	-0.006	0.020	0.019	93.67
Current value	α_{11}	0.02	0.020	0	0.005	0.005	96.32	0.020	0	0.005	0.005	96.33
Current slope	α_{21}	0.01	0.010	0	0.041	0.038	91.97	0.009	-0.001	0.041	0.039	93.67
Weibull	$\sqrt{\kappa_1}$	1.1	1.098	-0.002	0.036	0.038	95.99	1.097	-0.003	0.036	0.038	95.67
	ζ_{01}	-7	-6.934	0.066	0.703	0.722	96.32	-6.934	0.066	0.702	0.724	96.00
Survival submodel 2												
Current variance	$\alpha_{\sigma 2}$	0.15	0.154	0.004	0.022	0.020	93.31	0.154	0.004	0.021	0.021	95.33
Current value	α_{12}	-0.01	-0.010	0	0.005	0.005	95.65	-0.010	0	0.005	0.005	96.00
Current slope	α_{22}	-0.14	-0.149	-0.009	0.050	0.042	92.98	-0.148	-0.008	0.044	0.043	97.00
Weibull	$\sqrt{\kappa_2}$	1.3	1.304	0.004	0.048	0.047	95.65	1.303	0.003	0.048	0.047	95.67
	ζ_{02}	-4	-4.123	-0.123	0.765	0.776	93.65	-4.124	-0.124	0.763	0.786	95.00

ESE : Empirical Standard Error ; ASE : Asymptotic Standard Error ; Coverage rate : coverage rate of the 95% confidence interval.
* Results for 299 replicates with complete convergence over 300 for step 1 and 300 replicates over 300 for step 2.

Table S3 – Simulation results for scenario C with 1000 subjects (7 measures, b_i and τ_i correlated).*

**Parameter**			**Step 1 (S1 = 500 QMC)**					**Step 2 (S2 = 5000 QMC)**				
		**True value**	**Mean**	**Bias**	**ESE**	**ASE**	**CR (%)**	**Mean**	**Bias**	**ESE**	**ASE**	**CR (%)**
Longitudinal submodel												
Intercept	β_0	142	141.9	-0.1	0.564	0.529	93.31	141.9	-0.1	0.554	0.535	94.00
Slope	β_1	3	3.006	0.006	0.215	0.202	93.98	3.002	0.002	0.206	0.210	95.67
Variability	μ_0	2.4	2.402	0.002	0.024	0.023	93.65	2.400	0	0.024	0.023	95.33
	μ_1	0.05	0.049	-0.001	0.012	0.011	93.98	0.050	0	0.011	0.011	95.33
$\Sigma_{b\tau}$	$\sigma^2_{b_0}$	210.25	211.1	0.85	13.86	12.24	90.64	210.7	0.45	13.26	12.65	93.33
	$\sigma_{b_0b_1}$	-15.95	-15.72	0.23	3.211	2.904	92.64	-15.87	0.08	3.053	2.998	94.67
	$\sigma_{b_0\tau_0}$	2.9	2.811	-0.089	0.399	0.369	92.64	2.847	-0.053	0.386	0.378	93.67
	$\sigma_{b_0\tau_1}$	-0.145	-0.144	0.001	0.175	0.152	90.64	-0.131	0.014	0.162	0.155	93.00
	$\sigma^2_{b_1}$	9.05	9.155	0.105	1.163	1.019	90.97	9.172	0.122	1.065	1.032	95.00
	$\sigma_{b_1\tau_0}$	-0.304	-0.288	0.016	0.120	0.113	90.97	-0.291	0.013	0.114	0.117	94.33
	$\sigma_{b_1\tau_1}$	0.067	0.064	-0.003	0.051	0.044	90.64	0.061	-0.006	0.047	0.046	91.33
	$\sigma^2_{\tau_0}$	0.1309	0.123	-0.0079	0.020	0.019	90.64	0.129	-0.0019	0.019	0.019	95.67
	$\sigma_{\tau_0\tau_1}$	-0.0206	-0.020	0.0006	0.007	0.006	92.64	-0.020	0.0006	0.007	0.007	96.67
	$\sigma^2_{\tau_1}$	0.0141	0.014	-0.0001	0.003	0.003	90.64	0.014	-0.0001	0.003	0.003	95.00
Survival submodel 1												
Current variance	$\alpha_{\sigma 1}$	0.07	0.066	-0.004	0.034	0.032	94.98	0.067	-0.003	0.033	0.031	94.00
Current value	α_{11}	0.02	0.021	0.001	0.008	0.007	92.64	0.021	0.001	0.008	0.007	92.67
Current slope	α_{21}	0.01	-0.004	-0.014	0.055	0.051	93.65	-0.005	-0.015	0.053	0.054	93.67
Weibull	$\sqrt{\kappa_1}$	1.1	1.092	-0.008	0.037	0.037	94.98	1.092	-0.008	0.037	0.039	95.00
	ζ_{01}	-7	-7.123	-0.123	0.960	0.883	94.31	-7.122	-0.122	0.959	0.902	94.33
Survival submodel 2												
Current variance	$\alpha_{\sigma 2}$	0.15	0.154	0.004	0.034	0.034	94.98	0.154	0.004	0.034	0.034	96.67
Current value	α_{12}	-0.01	-0.010	0	0.009	0.009	95.99	-0.010	0	0.009	0.009	96.67
Current slope	α_{22}	-0.14	-0.143	-0.003	0.061	0.056	94.31	-0.141	-0.001	0.061	0.057	94.67
Weibull	$\sqrt{\kappa_2}$	1.3	1.302	0.002	0.047	0.045	94.31	1.303	0.003	0.046	0.046	95.67
	ζ_{02}	-4	-4.107	-0.107	1.003	1.021	96.66	-4.098	-0.098	1.005	1.052	96.33

ESE : Empirical Standard Error ; ASE : Asymptotic Standard Error ; Coverage rate : coverage rate of the 95% confidence interval.

* Results for 299 replicates with complete convergence over 300 for step 1 and 300 replicates over 300 for step 2.

Table S4 – Simulation results for scenario D with 500 subjects (13 measures, b_i and τ_i correlated).*

**Parameter**			**Step 1 (S1 = 500 QMC)**					**Step 2 (S2 = 5000 QMC)**				
		**True value**	**Mean**	**Bias**	**ESE**	**ASE**	**CR (%)**	**Mean**	**Bias**	**ESE**	**ASE**	**CR (%)**
Longitudinal submodel												
Intercept	β_0	142	141.9	-0.1	0.825	0.746	91.33	141.9	-0.1	0.812	0.756	91.64
Slope	β_1	3	2.980	-0.20	0.327	0.282	89.67	2.993	-0.007	0.311	0.295	93.65
Variability	μ_0	2.4	2.400	0	0.034	0.033	93.67	2.398	-0.002	0.034	0.033	94.65
	μ_1	0.05	0.049	-0.001	0.016	0.015	93.33	0.049	-0.001	0.016	0.016	93.98
$\Sigma_{b\tau}$	$\sigma^2_{b_0}$	210.25	210.7	0.45	21.02	16.98	88.67	210.5	0.25	20.41	17.85	91.97
	$\sigma_{b_0 b_1}$	-15.95	-15.57	0.38	4.788	3.991	88.33	-15.70	0.25	4.509	4.281	90.97
	$\sigma_{b_0 \tau_0}$	2.9	2.882	-0.018	0.580	0.525	91.67	2.922	0.022	0.554	0.538	94.31
	$\sigma_{b_0 \tau_1}$	-0.145	-0.171	-0.026	0.235	0.211	93.00	-0.162	-0.017	0.222	0.222	94.31
	$\sigma^2_{b_1}$	9.05	9.196	0.146	1.912	1.412	84.00	9.164	0.114	1.664	1.497	91.97
	$\sigma_{b_1 \tau_0}$	-0.304	-0.314	-0.010	0.180	0.157	91.33	-0.307	-0.003	0.169	0.162	92.98
	$\sigma_{b_1 \tau_1}$	0.067	0.073	0.006	0.072	0.061	90.33	0.069	0.002	0.065	0.064	94.65
	$\sigma^2_{\tau_0}$	0.1309	0.124	-0.0069	0.030	0.026	88.00	0.129	-0.0019	0.030	0.027	91.97
	$\sigma_{\tau_0 \tau_1}$	-0.0206	-0.020	0.0006	0.011	0.009	84.00	-0.020	0.0006	0.011	0.010	89.97
	$\sigma^2_{\tau_1}$	0.0141	0.014	-0.0001	0.006	0.004	82.33	0.014	-0.0001	0.005	0.005	89.63
Survival submodel 1												
Current variance	$\alpha_{\sigma 1}$	0.07	0.069	-0.001	0.050	0.046	94.33	0.070	0	0.049	0.046	94.65
Current value	α_{11}	0.02	0.021	0.001	0.011	0.011	92.67	0.021	0.001	0.011	0.011	93.31
Current slope	α_{21}	0.01	0.013	0.003	0.086	0.076	94.00	0.011	0.001	0.082	0.076	95.32
Weibull	$\sqrt{\kappa_1}$	1.1	1.102	0.002	0.054	0.054	95.0	1.102	0.002	0.054	0.053	94.65
	ζ_{01}	-7	-7.170	-0.170	1.389	1.289	93.33	-7.178	-0.178	1.379	1.292	93.98
Survival submodel 2												
Current variance	$\alpha_{\sigma 2}$	0.15	0.184	0.034	0.196	0.057	93.0	0.176	0.026	0.167	0.063	95.99
Current value	α_{12}	-0.01	-0.017	-0.007	0.049	0.015	89.67	-0.015	-0.005	0.041	0.016	93.31
Current slope	α_{22}	-0.14	-0.170	-0.030	0.238	0.093	96.67	-0.167	-0.027	0.330	0.104	97.32
Weibull	$\sqrt{\kappa_2}$	1.3	1.326	0.026	0.157	0.072	93.00	1.324	0.024	0.164	0.075	93.98
	ζ_{02}	-4	-3.697	0.303	3.413	1.643	91.00	-3.817	0.183	2.702	1.718	92.46

ESE : Empirical Standard Error ; ASE : Asymptotic Standard Error ; Coverage rate : coverage rate of the 95% confidence interval.

* Results for 300 replicates with complete convergence over 300 for step 1 and 299 replicates over 300 for step 2.

Table S5 – Simulation results for scenario D with 1000 subjects (13 measures, b_i and τ_i correlated).*

**Parameter**			**Step 1 (S1 = 500 QMC)**					**Step 2 (S2 = 5000 QMC)**				
		**True value**	**Mean**	**Bias**	**ESE**	**ASE**	**CR (%)**	**Mean**	**Bias**	**ESE**	**ASE**	**CR (%)**
Longitudinal submodel												
Intercept	β_0	142	141.9	-0.1	0.575	0.532	91.30	141.9	-0.1	0.557	0.540	93.67
Slope	β_1	3	3.003	0.003	0.209	0.202	93.31	3.007	0.007	0.197	0.209	95.67
Variability	μ_0	2.4	2.405	0.005	0.025	0.023	93.65	2.402	0.002	0.025	0.023	94.33
	μ_1	0.05	0.049	-0.001	0.012	0.011	93.31	0.049	-0.001	0.011	0.011	94.33
$\Sigma_{b\tau}$	$\sigma^2_{b_0}$	210.25	212.6	2.3	14.51	12.35	91.97	212.5	2.2	13.32	12.88	94.00
	$\sigma_{b_0 b_1}$	-15.95	-15.61	0.34	3.440	2.883	91.30	-15.87	0.08	3.064	3.024	94.33
	$\sigma_{b_0 \tau_0}$	2.9	2.856	-0.044	0.417	0.370	90.97	2.898	-0.002	0.404	0.382	92.67
	$\sigma_{b_0 \tau_1}$	-0.145	-0.169	-0.024	0.171	0.150	90.97	-0.151	-0.006	0.161	0.157	93.67
	$\sigma^2_{b_1}$	9.05	9.061	0.011	1.298	1.021	89.97	9.123	0.073	1.049	1.066	94.67
	$\sigma_{b_1 \tau_0}$	-0.304	-0.306	-0.002	0.124	0.112	92.31	-0.304	0	0.116	0.115	94.33
	$\sigma_{b_1 \tau_1}$	0.067	0.073	0.006	0.049	0.043	92.31	0.068	0.001	0.045	0.044	95.33
	$\sigma^2_{\tau_0}$	0.1309	0.123	-0.0079	0.021	0.018	90.97	0.130	-0.0009	0.020	0.020	93.33
	$\sigma_{\tau_0 \tau_1}$	-0.0206	-0.019	0.0016	0.008	0.006	89.63	-0.208	-0.1874	0.007	0.007	94.33
	$\sigma^2_{\tau_1}$	0.0141	0.013	-0.0011	0.004	0.003	86.29	0.014	-0.0001	0.003	0.003	95.00
Survival submodel 1												
Current variance	$\alpha_{\sigma 1}$	0.07	0.063	-0.007	0.035	0.031	91.30	0.065	-0.005	0.034	0.031	94.00
Current value	α_{11}	0.02	0.021	0.001	0.008	0.007	93.65	0.021	0.001	0.008	0.007	94.00
Current slope	α_{21}	0.01	0.014	0.004	0.053	0.051	94.31	0.015	0.005	0.051	0.050	94.67
Weibull	$\sqrt{\kappa_1}$	1.1	1.099	-0.001	0.035	0.037	95.32	1.099	-0.001	0.034	0.037	96.33
	ζ_{01}	-7	-7.087	-0.087	0.850	0.860	96.66	-7.087	-0.087	0.853	0.867	96.33
Survival submodel 2												
Current variance	$\alpha_{\sigma 2}$	0.15	0.159	0.009	0.042	0.035	92.64	0.159	0.009	0.040	0.035	95.00
Current value	α_{12}	-0.01	-0.012	-0.002	0.011	0.009	94.65	-0.012	-0.002	0.010	0.009	94.67
Current slope	α_{22}	-0.14	-0.151	-0.011	0.074	0.060	92.98	-0.150	-0.010	0.063	0.058	95.67
Weibull	$\sqrt{\kappa_2}$	1.3	1.313	0.013	0.046	0.046	94.98	1.313	0.013	0.047	0.046	95.00
	ζ_{02}	-4	-3.938	0.062	1.160	1.041	94.31	-3.937	0.063	1.156	1.054	94.67

ESE : Empirical Standard Error ; ASE : Asymptotic Standard Error ; Coverage rate : coverage rate of the 95% confidence interval.

* Results for 300 replicates with complete convergence over 300.

Table S6 – Simulation results for scenario E with 500 subjects (Misspecified model : quadratic time trend in the data generation model vs linear time-trend in the estimated model).*

**Parameter**			**Step 1 (S1 = 500 QMC)**					**Step 2 (S2 = 5000 QMC)**				
		True Value	**Mean**	**Bias**	**ESE**	**ASE**	**CR (%)**	**Mean**	**Bias**	**ESE**	**ASE**	**CR (%)**
Longitudinal submodel												
Intercept	β_0	142	140.8	-1.2	0.808	0.667	52.67	140.8	-1.2	0.791	0.701	56.67
Slope	β_1	0.7	3.129	2.429	0.208	0.190	0	3.135	2.435	0.202	0.192	0
Variability	μ_0	2.4	2.406	0.006	0.024	0.023	94.00	2.403	0.003	0.023	0.024	95.67
	μ_1	0.05	0.052	0.002	0.011	0.011	92.00	0.052	0.002	0.011	0.011	93.00
Σ_b	$\sigma^2_{b_0}$	210.25	201.1	-9.15	18.24	14.41	78.33	200.9	-9.35	17.80	15.48	81.67
	$\sigma_{b_0b_1}$	-17.4	-5.874	11.526	3.567	2.986	8.33	-6.096	11.304	3.308	3.097	8.67
	$\sigma^2_{b_1}$	9.28	7.067	-2.213	1.372	0.991	41.33	7.068	-2.212	1.203	1.027	43.67
Σ_τ	$\sigma^2_{\tau_0}$	0.09	0.085	-0.005	0.019	0.016	88.00	0.090	0	0.018	0.016	92.33
	$\sigma_{\tau_0\tau_1}$	-0.018	-0.016	0.002	0.007	0.006	86.67	-0.018	0	0.007	0.006	93.00
	$\sigma^2_{\tau_1}$	0.0136	0.012	-0.0016	0.003	0.003	83.67	0.014	0.0004	0.003	0.003	92.00
Survival submodel												
Current variance	α_σ	0	-0.001	-0.001	0.023	0.022	96.00	0.001	0.001	0.022	0.022	95.00
Current value	α_1	0.03	0.030	0	0.005	0.005	95.67	0.030	0	0.005	0.005	95.67
Weibull	$\sqrt{\kappa}$	1.1	1.108	0.008	0.041	0.040	94.00	1.108	0.008	0.041	0.040	94.00
	ζ_0	-7	-6.965	0.035	0.788	0.789	94.33	-6.966	0.034	0.786	0.787	94.33

ESE : Empirical Standard Error ; ASE : Asymptotic Standard Error ; Coverage rate : coverage rate of the 95% confidence interval.
* Results for 300 replicates with complete convergence over 300 for step 1 and step 2.

Table S7 – Simulation results for scenario F1 with 500 subjects : estimation of the model with time-dependent residual variance on data generated with time-fixed residual variance.*

**Parameter**			**Step 1 (S1 = 500 QMC)**					**Step 2 (S2 = 5000 QMC)**				
		**True value**	**Mean**	**Bias**	**ESE**	**ASE**	**CR (%)**	**Mean**	**Bias**	**ESE**	**ASE**	**CR (%)**
Longitudinal submodel												
Intercept	β_0	142	142.0	0	0.762	0.734	93.00	142.0	0	0.756	0.744	94.67
Slope	β_1	3	2.976	-0.024	0.230	0.223	93.00	2.977	-0.023	0.227	0.224	93.67
Variability	μ_0	2.4	2.398	-0.002	0.032	0.032	94.67	2.396	-0.004	0.033	0.032	94.33
	μ_1	0	0.0006	0.0006	0.013	0.013	95.0	0.001	0.001	0.013	0.013	95.67
Σ_b	$\sigma^2_{b_0}$	210.25	214.5	4.25	19.91	17.27	90.27	214.0	3.75	19.59	17.66	92.33
	$\sigma_{b_0b_1}$	-15.95	-16.28	-0.33	4.31	3.75	90.67	-16.41	-0.46	4.13	3.81	93.67
	$\sigma^2_{b_1}$	9.05	9.012	-0.038	1.322	1.276	93.67	9.078	0.028	1.251	1.290	95.33
Σ_τ	$\sigma^2_{\tau_0}$	0.09	0.097	0.007	0.024	0.024	95.0	0.096	0.006	0.024	0.024	95.67
	$\sigma_{\tau_0\tau_1}$	0	-0.004	-0.004	0.006	0.006	93.00	-0.003	-0.003	0.006	0.007	95.33
	$\sigma^2_{\tau_1}$	0	0.002	0.002	0.002	0.002	96.00	0.002	0.002	0.002	0.002	96.67
Survival submodel 1												
Current variance	$\alpha_{\sigma 1}$	0.07	0.073	0.003	0.047	0.046	97.67	0.073	0.003	0.046	0.044	97.0
Current value	α_{11}	0.02	0.020	0	0.008	0.007	94.67	0.020	0	0.007	0.007	94.00
Current slope	α_{21}	0.01	0.004	-0.006	0.068	0.065	95.0	0.004	-0.006	0.067	0.064	95.67
Weibull	$\sqrt{\kappa_1}$	1.1	1.106	0.006	0.050	0.052	97.0	1.107	0.007	0.050	0.052	97.0
	ζ_{01}	-7	-7.058	-0.058	1.327	1.278	94.33	-7.058	-0.058	1.327	1.274	94.33

ESE : Empirical Standard Error ; ASE : Asymptotic Standard Error ; Coverage rate : coverage rate of the 95% confidence interval.
* Results for 300 replicates with complete convergence over 300.

TABLE S8 – Simulation results for scenario F2 with 500 subjects : estimation of the model with time-fixed residual variance on data generated with time-fixed residual variance.*

**Parameter**			**Step 1 (S1 = 500 QMC)**					**Step 2 (S2 = 5000 QMC)**				
		**True value**	**Mean**	**Bias**	**ESE**	**ASE**	**CR (%)**	**Mean**	**Bias**	**ESE**	**ASE**	**CR (%)**
Longitudinal submodel												
Intercept	β_0	142	142.1	0.1	0.770	0.732	94.33	142.0	0	0.765	0.741	93.67
Slope	β_1	3	2.973	-0.027	0.232	0.224	93.33	2.974	-0.026	0.227	0.224	93.67
Variability	μ_0	2.4	2.403	0.003	0.023	0.023	94.67	2.400	0	0.024	0.023	93.33
Σ_b	$\sigma^2_{b_0}$	210.25	214.3	4.05	19.71	17.24	93.00	213.84	3.59	19.43	17.61	93.33
	$\sigma_{b_0 b_1}$	-15.95	-16.35	-0.40	4.12	3.75	93.33	-16.46	-0.51	4.04	3.78	93.67
	$\sigma^2_{b_1}$	9.05	9.093	0.043	1.300	1.284	92.67	9.128	0.078	1.244	1.270	95.67
Σ_τ	$\sigma^2_{\tau_0}$	0.09	0.089	-0.001	0.014	0.014	93.33	0.090	0	0.014	0.014	95.00
Survival submodel 1												
Current variance	$\alpha_{\sigma 1}$	0.07	0.069	-0.001	0.044	0.044	96.67	0.070	0	0.043	0.043	97.33
Current value	α_{11}	0.02	0.020	0	0.007	0.007	95.67	0.020	0	0.007	0.007	95.33
Current slope	α_{21}	0.01	0.003	-0.007	0.069	0.064	94.33	0.004	-0.006	0.066	0.064	96.00
Weibull	$\sqrt{\kappa_1}$	1.1	1.108	0.008	0.048	0.051	97.00	1.108	0.008	0.048	0.051	97.33
	ζ_{01}	-7	-7.009	-0.009	1.306	1.260	95.00	-7.009	-0.009	1.305	1.262	94.33

ESE : Empirical Standard Error ; ASE : Asymptotic Standard Error ; Coverage rate : coverage rate of the 95% confidence interval.
* Results for 300 replicates with complete convergence over 300.

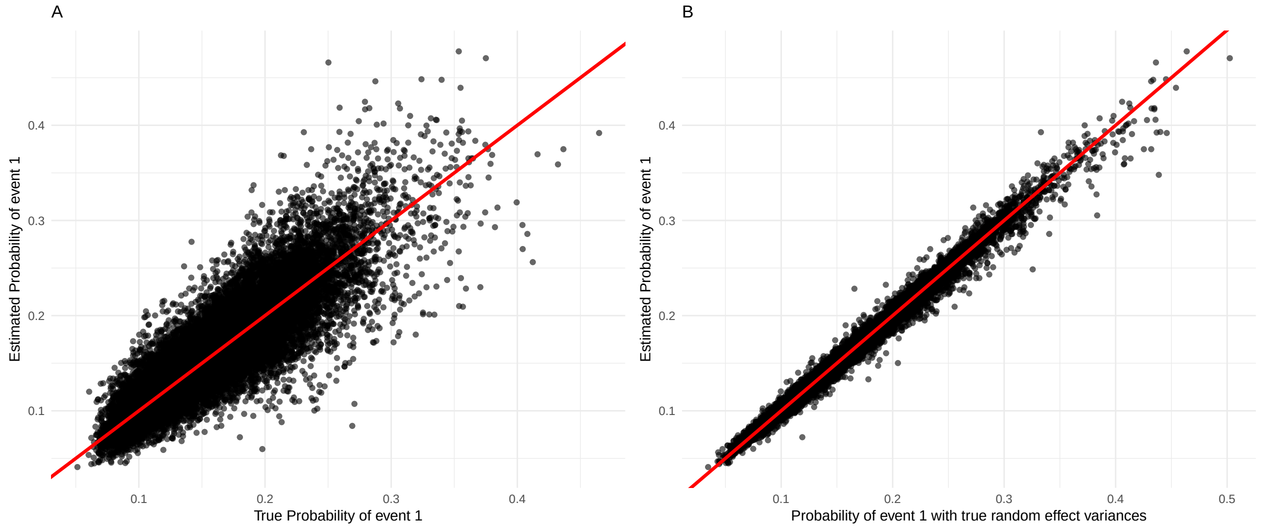

Figure S1 – Comparison across 100 replications from Scenario B of individual estimated probabilities of event 1 (at 5 years given event free at 3 years and measurements collected up to 3 years) with the true probabilities computed using the model parameters (Panel A), and with the probabilities computed using the parameters estimated by the model except for the parameters of the random effects covariance matrix, where the true parameter values where considered (panel B).

Table S9 – Parameter estimates of the CVCS joint model on the Progress clinical trial data.

**Parameter**	**Estimate**	**Standard error**	**p-value**
Survival submodel for CVD			
BP current value	-0.009	0.004	0.024
BP current slope	-0.0743	0.015	< 0.001
BP0	0.006	0.003	0.015
treatment group	-0.204	0.084	0.015
male	0.270	0.088	0.002
age	0.043	0.005	0.002
Survival submodel for Death			
BP current value	-0.013	0.011	0.222
BP current slope	-0.135	0.046	0.003
BP0	-0.002	0.006	0.702
treatment group	-0.013	0.325	0.969
male	0.474	0.193	0.014
age	0.057	0.010	< 0.001
Longitudinal submodel			
Blood Pressure Mean			
intercept	143.3	0.392	< 0.001
time	-0.018	0.336	0.958
time^2	-0.151	0.081	0.061
treatment group	-9.553	0.549	< 0.001
time $\times$ treatment group	1.088	0.444	0.014
time $\times$ treatment group^2	-0.072	0.107	0.500
Blood Pressure Residual Variance			
intercept	2.321	0.012	< 0.001
time	0.007	0.004	0.088
treatment group	-0.033	0.014	0.018

BP : Blood Pressure, BP0 : Blood pressure at inclusion

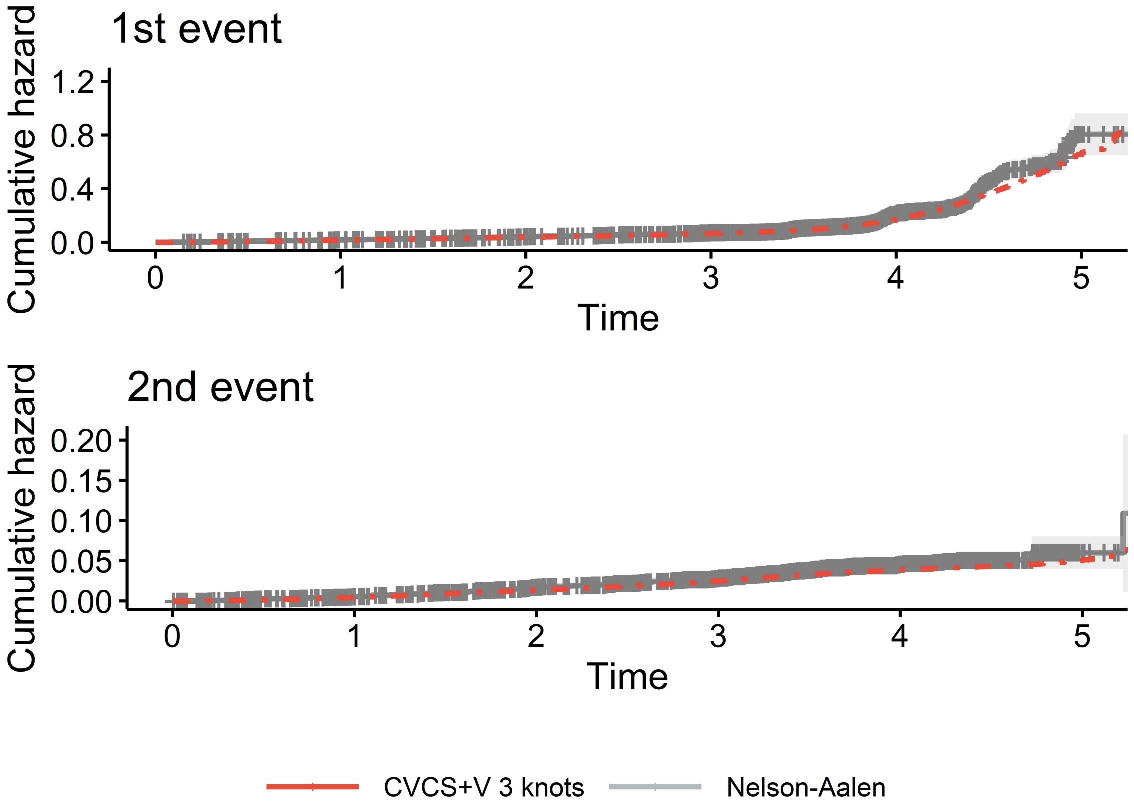

FIGURE S2 – Survival submodel for CVD (top) and death (bottom) fit assessment : comparison between predicted cumulative hazard function (in purple) and Nelson Aalen estimator (in grey).

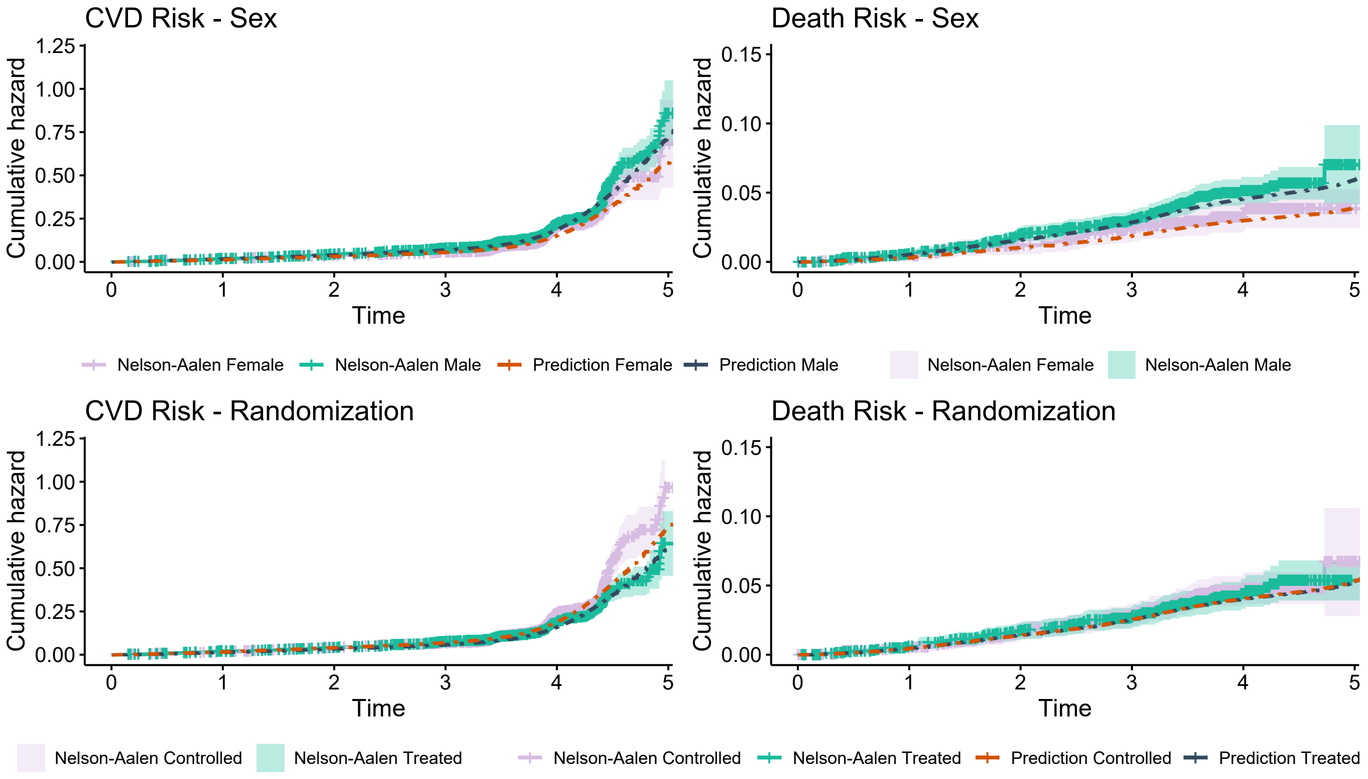

FIGURE S3 – Survival submodel for CVD (left) and death (right) fit assessment for Sex (top) and Randomization group (bottom) : comparison between predicted cumulative hazard function and Nelson Aalen estimator.

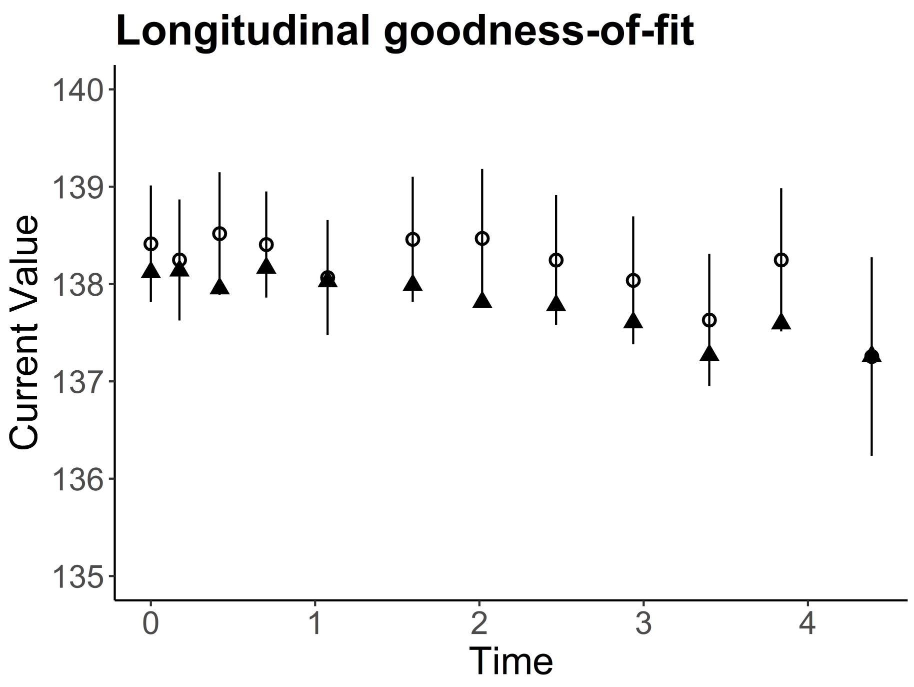

FIGURE S4 – Mixed effects submodel : Comparison between predicted value of the marker from the joint model (black triangles) and the observations (mean in white circles with 95% confidence interval).
